# Polymer-Based Hydrogels for Chemical Biosensing: From Bioreceptor Integration to Smart Applications

**DOI:** 10.1021/acsapm.6c02489

**Published:** 2026-09-28

**Authors:** Leyre De-La-Fuente, Miryam Criado-Gonzalez, Bergoi Ibarlucea

**Affiliations:** † TECNALIA, Basque Research and Technology Alliance (BRTA), Mikeletegi Pasealekua 2, Donostia-San Sebastián 20009, Spain; ‡ Departments of Applied Chemistry and Polymers and Advanced Materials: Physics, Chemistry and Technology, Faculty of Chemistry, University of the Basque Country (EHU), 20018 Donostia-San Sebastián, Spain; § Institute of Polymer Science and Technology, CSIC, Juan de la Cierva 3, Madrid 28006, Spain

**Keywords:** functional hydrogels, conductive polymers, hydrogel-based biosensors, bioreceptor immobilization, biosensing

## Abstract

In recent years, flexible, wearable, and implantable chemical sensors and biosensors have become crucial in biomedical engineering. These flexible devices enable the noninvasive, real-time monitoring of biochemical markers in biofluids, from blood to the most accessible ones that enable noninvasive monitoring (sweat, tears, saliva, and interstitial fluid) as indicators of the health status. In their development, polymer-based hydrogels have emerged as the material of choice and have recently started to attract increasing attention due to advantages in terms of biocompatibility, surface area, and chemical tunability. Here, we provide an overview of the structural polymers according to their nature (natural vs synthetic), giving a special focus on the use of conductive polymers. Then, we discuss the strategies to integrate the bioreceptor to provide the hydrogels with the necessary selectivity toward biomarker detection. Finally, we go through emerging trends where hydrogels are employed in smart formats beyond the simple performance of the detection. As an overall, the integration of polymer-based hydrogels with advanced bioreceptor strategies and smart functionalities is driving the development of next-generation wearable and implantable biosensors, paving the way for noninvasive, continuous, and personalized diagnostics.

## Graphical abstract

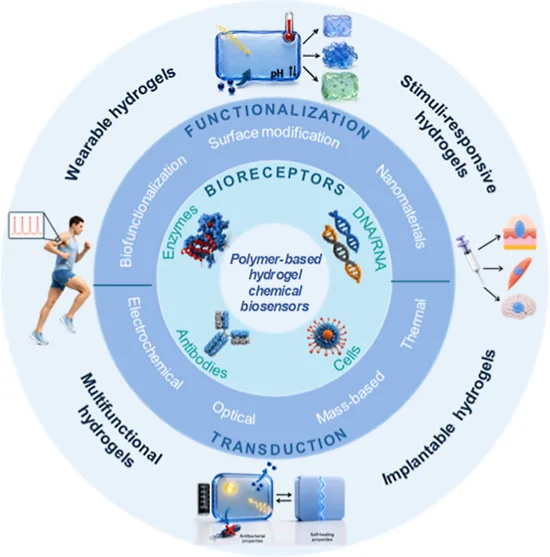

## 1 Introduction

The development of rapid, ultrasensitive, and portable biosensors as medical devices has revolutionized the field of early disease diagnosis and monitoring, which is essential for patient survival and successful prognosis.^1,2^ Currently, the demand for biosensors has grown substantially, driven by their integration in therapeutic interventions and patient care. These technologies have transformed traditional healthcare practices, reducing reliance on time-consuming clinical examinations and invasive diagnosis procedures.^3^ For instance, the successful development of rapid point-of-care (POC) diagnostic tests for the early detection of infectious Corona Virus diseases during the global COVID-19 pandemic proved the urgency for searching new effective diagnostic tools.^2,4^ As a result, research into innovative biosensor technologies such as wearable biosensors, implantable devices, and POC platforms has grown significantly.^5–9^

The human body consists of soft and high-water containing tissues and organs.^10^ Despite remarkable advances in the recent years, almost all commercially available and most lab-level bioelectronic devices are composed of rigid, dry, and synthetic electronic materials.^11^ These disparities between living biological tissues and electronic systems have introduced several challenges in the development of biosensors.^10^ As an example, some medical-grade wearable sensors, such as those employing conventional Ag/AgCl electrodes, may cause irritation and allergic reactions.^12^ Moreover, considering the higher level of various biomarkers in blood than in other biofluids such as tears, saliva, sweat and interstitial fluid (ISF), several current biosensors involve invasive sampling techniques.^13^ Therefore, the growing preference for noninvasive methods and less cytotoxic materials has created a demand for new biomaterials for biological and chemical sensing for diagnostics.^14^ Among the various technologies appearing, functional polymer-based hydrogels are gaining increasing interest as a powerful tool for the design and fabrication of flexible biosensing devices (Figure 1a).^15–18^ In fact, the numbers of total publications related to hydrogel biosensors and hydrogel-based flexible biosensors research has increased significantly over the last 10 years, tripling in the last five years, according to the Web of Science^19^ and Scopus (Figure 1b).

**Figure 1:**
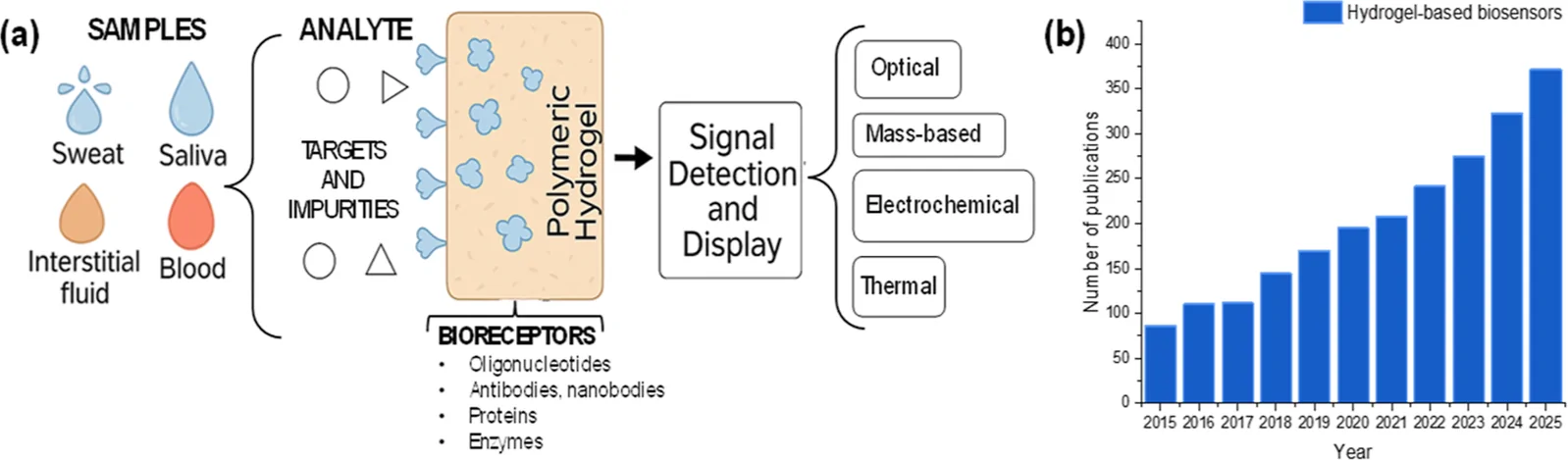
Hydrogel-based biosensors: (a) Schematic representation of a polymeric hydrogel biosensing platform. Saliva, blood, interstitial fluid, and sweat are used as biological samples containing target analytes and potential impurities. The specific analytes interact with bioreceptors immobilized within a polymeric hydrogel matrix, where biochemical recognition occurs. The biochemical event is converted into a measurable signal, which is subsequently processed for signal detection and display. (b) Number of publications about hydrogel-based biosensors. Data obtained from Scopus.^20^

Hydrogels are three-dimensional (3D) cross-linked networks adept at retaining substantial volumes of biocompatible liquids.^21^ In contrast to materials conforming traditional biosensors, hydrogels possess unique properties that make them highly suitable for continuous, in vivo assessments. Compared to 2D planar surfaces, hydrogels present a versatile and dynamic 3D network, high biocompatibility, flexibility, tunability, favorable mechanical properties, easy synthesis, and specificity, among other advantages.^22^ Due to their resemblance to natural biological soft tissues, hydrogels have been extensively studied in the biomedical field. Initial studies in the field of hydrogels for biosensing applications explored the potential of hydrophilic polymers, driven by their unique ability to retain water and mimic biological environments such as the extracellular matrix or mucus.^23–25^ Moreover, early in vivo studies focused on implantable hydrogel systems and have played a crucial role in assessing their biocompatibility and sensing accuracy.^26–28^ Hydrogel-based biosensors have also been proved to be economically sustainable by employing simple fabrication methods and readily available, inexpensive materials.^19^

Particularly, polymers are ideal candidates for biosensing applications due to their versatile chemical structures, tunable physical properties, and ease of functionalization (Figure 2).^29^ These characteristics allow the design of materials with specific mechanical, optical, and electrical properties, as well as higher tissue compatibility and biosafety. In addition, conductive polymers are recently regarded as the most promising materials in bioelectronics due to their unique electrochemical properties, enabling their integration into electrochemical biosensors,^30^ which has opened new frontiers in the medical technology, especially for wearable and implantable sensors.^31–34^

**Figure 2:**
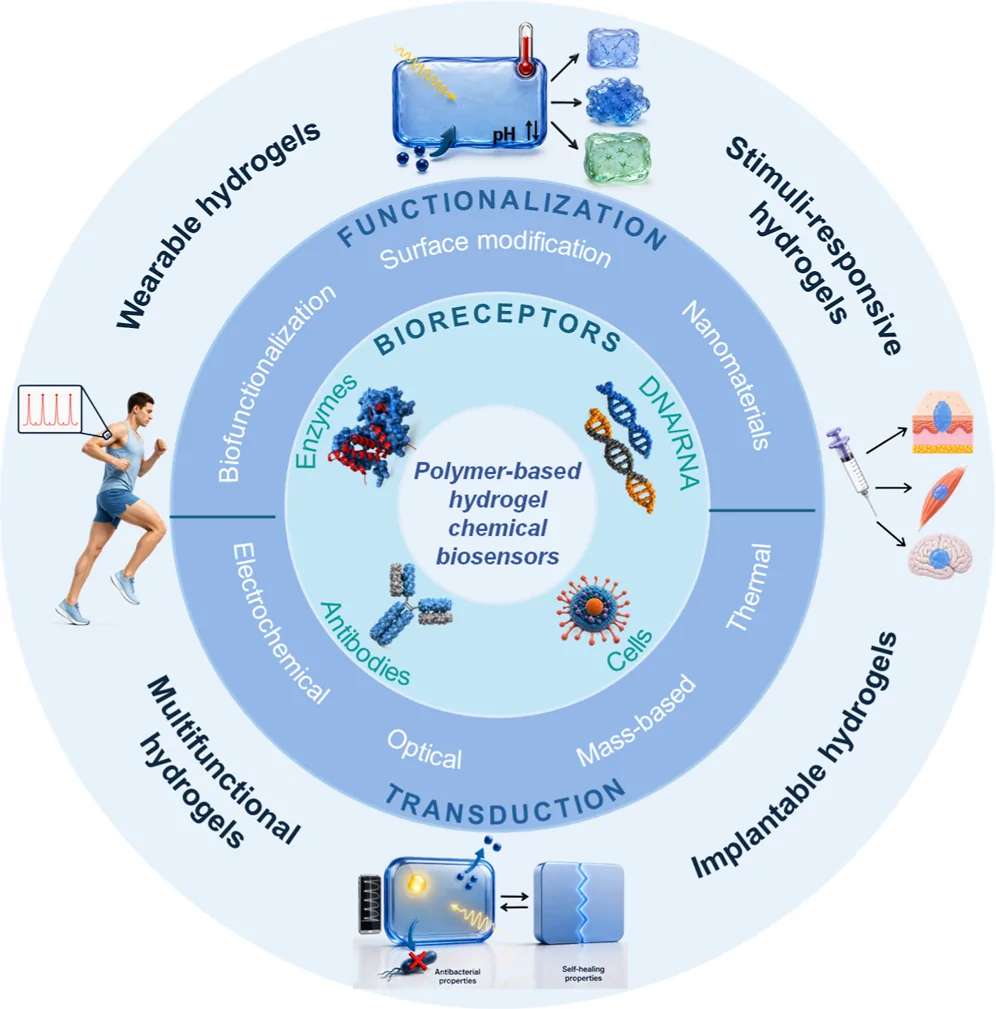
Schematic illustration of the design and functional roles played by polymer-based hydrogel chemical biosensors.

Despite their unique and exceptional properties, several studies have demonstrated that polymer-based hydrogels may have some limitations. Hydrogels generally show a swelling–weakening behavior that reduces polymer chain network density, which results in a decline in mechanical strength and poor processability.^35–37^ Long-term stability has also appeared to be a challenge, as they may degrade over time, requiring careful storage and handling.^38^ The immobilization and surface bonding of bioreceptors on hydrogels may be challenging, and some materials may have potential for nonspecific binding, leading to false positives if not carefully optimized.^39,40^ Finally, in the specific context of conductive hydrogels, commonly used polymers intrinsically have very low electrical conductivity,^22,41^ limiting the choice of materials or requiring further modifications to produce hydrogels with good electrical properties. Therefore, despite the evidential progress in polymer-based hydrogels for biosensing, several challenges still remain.

Several recent reviews have discussed hydrogel-based biosensors from perspectives such as hydrogel fabrication and stimuli-responsive behavior, wearable and implantable sensing platforms, and specific hydrogel classes like aptamer-functionalized systems.^42–44^ These reviews are generally organized around material properties, sensing mechanisms, device configurations, or individual recognition elements, rather than providing a dedicated discussion of bioreceptor integration strategies across diverse polymer-based hydrogel platforms. In this context, this review article provides a comprehensive overview of recent advances and emerging applications of polymer-based hydrogels in chemical biosensing technologies and complements the existing literature by focusing on the incorporation of antibodies, aptamers, enzymes, nucleic acids, and other recognition elements into hydrogel matrices. The review discusses the structural and functional properties of polymer hydrogels, recent developments in hydrogel functionalization and bioreceptor immobilization, and their impact on biosensor selectivity, sensitivity, and analytical performance. Special attention is given to conductive hydrogels and emergent smart biosensing platforms, highlighting the links between hydrogel design, molecular recognition, and signal transduction. Finally, it addresses the current challenges and outlines future research directions aimed at optimizing the design of next-generation polymer-based hydrogels for biosensing applications in clinical diagnosis.

## 2 Polymer-Based Hydrogels for Chemical Biosensing

Polymer-based hydrogels can be divided into two categories depending on the origin of the precursors: those obtained from natural polymers (e.g., agarose, chitosan, alginate, etc.), and hydrogels composed of synthetic polymers (e.g., polyethylene glycol (PEG), poly­(2-hydroxylethyl methacrylate) (PHEMA), etc.).^34^ This division has soft limits, being possible to find hybrid or semisynthetic examples.^45^ Moreover, hydrogels made of conductive polymers is a special category worthy to mention, opening new possibilities independently on being natural or synthetic.^46^ These materials contain multiple functional groups that allow further physicochemical modifications to provide networks with additional functional properties.^47^ Depending on the cross-linking mechanism, the hydrogels can be classified into chemical hydrogels, if polymer chains are cross-linked through chemical covalent bonds (simultaneously or post polymerization), and physical hydrogels when the cross-linking takes place via coordinative, electrostatic, hydrophobic, and dipole–dipole interactions.^41,48^ Moreover, dual-network hydrogels can also be obtained by the combination of physical and chemical cross-linked polymer networks.^49,50^ Interestingly, polymer-based hydrogels can also exhibit stimuli-responsive behavior. These stimuli include physical triggers (e.g., temperature, light, magnetic or electric fields, etc.), chemical cues (e.g., pH, redox balance, etc.), and biochemical signals (e.g., enzymes, glucose, etc.).^49,51^ In response to such stimuli, hydrogels can undergo changes in properties such as volume, color, swelling ratio, viscosity, conductivity, or permeability.^52^ For instance, alginate/PVA hydrogel-based colorimetric biosensors were developed for uric acid detection (2–600 μM), with a low detection limit of 1.92 μM and a rapid response time of 15 min^53^ Notably, electrosensitive hydrogels have recently attracted significant interest owing to their considerable potential in biosensing, flexible wearable technologies, and drug delivery applications, among others.^41,54^

Despite their significant potential, polymer-based hydrogels still face challenges, such as limited mechanical stability, slow analyte diffusion, loss of bioreceptor activity over time, and difficulties in large-scale manufacturing and device integration. To overcome these limitations, current research focuses on advanced hydrogel designs, including nanocomposite, double-network, and conductive hydrogels, which improve the mechanical properties, sensitivity, and signal transduction. In addition, emerging fabrication techniques such as 3D printing and microfabrication are expected to enhance the reproducibility and facilitate the development of robust hydrogel-based biosensors for real-world and POC applications.

### 2.1 Natural Polymer-Based Hydrogels

Biosensors require materials that exhibit high biocompatibility to ensure accurate in vivo applications. Unfortunately, many nonhydrogel-based commercially available biosensors do not fulfill this requirement.^11^ To overcome this limitation, natural polymers (i.e., cellulose, alginate, gelatin, etc.) have emerged as promising alternatives due to their inherent biocompatibility, biodegradability, and tunable physicochemical properties, making them suitable for biosensing applications that involve direct contact with the skin, such as wearable sensors for healthcare applications.^34,55^

Cellulose, the most abundant natural polymer, possesses intrinsic hydrophilicity, mechanical flexibility, and the ability to form 3D structures, which enable the formation of hydrogels.^56^ Cellulose and its derivatives exhibit high biocompatibility, making them promising candidates for the incorporation of biologically active substances, such as biomarkers.^57^ Silva et al.^58^ focused on the development of a skin-interfaced electrochemical biosensor based on microbial nanocellulose (MNC) for noninvasive monitoring of biomolecules in sweat. They fabricated a screen-printed carbon electrode on MNC and demonstrated its ability to detect various analytes across physiologically relevant concentrations, including toxic metals (low detection limit (LOD) of 4.5–14.5 μM for Cd^2+^ and 1.0–10.5 μM for Pb^2+^), uric acid (LOD of 1.8–11 μM), and 17β-estradiol (LOD of 0.27–0.58 μM). This approach represents a promising strategy for the accurate and real-time detection of sweat biomarkers, underscoring its potential utility in personalized healthcare and the continuous monitoring of diverse disease conditions. Lignin is another naturally derived polymer and is considered the second most abundant biopolymer after cellulose. Owing to its polyphenolic structure, lignin exhibits intrinsic antioxidant and antimicrobial properties, making it an attractive component for the development of functional hydrogel systems for biomedical and sensing applications.^59^ Winters et al.^60^ developed a lignin-reinforced HA/PAA hybrid hydrogel incorporating conductive nanoparticles, which exhibited low cytotoxicity (>80% cell viability), high adhesion strength (>200 N m^–1^), a Young’s modulus of up to 85 kPa, and swelling ratios approaching 9700%. These properties highlight the potential of lignin-based hydrogels for bioelectronic and biosensing applications. Moreover, hydrogels based on lignin and poly­(ethylene glycol (EG)) diglycidyl ether (PEGDGE) have exhibited high conductivity (5.87 S m^–1^) and demonstrated considerable potential as ionic thermoelectric materials. Silk fibroin,^61^ produced by silkworms such as *Bombyx mori* or spiders, has also emerged as a promising natural building block for a wide range of applications, particularly for multifunctional conductive hydrogels due to its excellent biocompatibility, abundant functional groups, and ability to form reversible β-sheet structures that enhance mechanical performance.^62,63^ A multifunctional SF-based hydrogel was developed in which the protein network provided mechanical reinforcement through hydrogen bonding, electrostatic interactions, and sacrificial β-sheet domains. The resulting hydrogel exhibited outstanding stretchability (up to 1390% strain), high tensile strength (0.60 MPa), and high ionic conductivity (2.41 S m^–1^), enabling its application as a wearable strain sensor with a wide sensing range of up to 700%.^64^

Agarose is another biocompatible, inexpensive, transparent and nontoxic polymer that can retain a high amount of water, resulting in high ionic conductivity. For instance, Lee et al.^65^ developed a transparent and ion-conductive agarose-based hydrogel, which was doped with NaCl and sucrose to provide ionic conductivity and enhance the water retention properties, respectively. This hydrogel exhibited excellent mechanical properties (Young’s modulus value close to 0.3 MPa), ionic conductivity (1.6 × 10^–2^ S cm^–1^), and self-healing ability (the electrical current was restored after recontacting two pieces of gel which were previously cut), when compared to traditional agarose hydrogels. The further fabrication of a transparent pressure sensor based on this hydrogel showed that the sensor could rapidly detect variations in applied pressure (8 and 15 kPa) through changes in hydrogel capacitance. Moreover, Shao et al.^66^ developed an integrated wearable flexible hydrogel patch for the noninvasive detection of physiological markers such as glucose, lactate, and ethanol in sweat. The system incorporated an agarose hydrogel to encapsulate biological oxidative enzymes, silver nanowires-dopamine (Ag NWs-PB) as the signal generation module, and a color-changing 3,3′, 5,5′-tetramethylbenzidine (TMB) probe as the signal output module. This hydrogel-based system was then combined with sweat-collectible (polydimethylsiloxane-silicon dioxide) PDMS-SiO_2_ patches. Glucose oxidase (GOx) was encapsulated, which allowed the rapid and accurate detection of glucose in sweat within 3.5 min, with a linear range of 0.02–5 mmol/L and an LOD of 0.011 mmol/L (Figure 3a). Chitosan (corn starch (CS)) is another natural polymer that exhibits good biocompatibility, biodegradability, adhesive potential, and antioxidant activity. It has gained interest for improving the conformality and mechanical properties of hydrogels in biosensing and drug delivery applications.^67^ It is a promising candidate for enhancing the interfacial adhesion between hydrogel sensors and human skin to improve the accuracy of signal monitoring.^68^ For instance, Li et al.^69^ developed an adhesive chitosan-based ionic conductive hydrogel by forming a physically cross-linked network with Al^3+^ ions that improved not only the ionic conductivity (up to 5.01 S/m) but also the mechanical performance (high stretchability up to 1000%, toughness of 730 kJ/m^3^, and good compression performance). The resulting flexible sensor demonstrated high sensitivity for detecting large human motion, demonstrated by elbow, wrist, and knee joint measures. In addition, Ye et al.^70^ constructed a surface-enhanced Raman scattering (SERS) aptasensor for rapid and sensitive detection of *Escherichia coli* (*E. coli*) in foods. In particular, SERS exploits the capability of metallic nanostructures to concentrate electromagnetic energy to achieve the detection of food-borne pathogens.^71^ The biosensor was composed of a CS-based hydrogel modified with *E. coli* aptamer (Apt)-functionalized silver nanoparticles (AgNPs) as a capture probe (Apt-AgNPs-CS gel). After incubation of the hydrogel in the presence of *E. coli*, the Raman signaling molecule 4-mercaptobenzoic acid (4-MBA) and *E. coli* Apt modified gold nanostars (GNS) (GNS@4-MBA-Apt NPs) as a signal probe for the sensitive quantification of *E. coli* (Figure 3b). The SERS aptasensor successfully detected *E. coli* with excellent selectivity and with an LOD as low as 3.46 CFU/mL in a linearized detection range (3.2 × 10^1^ to 3.2 × 10^7^ CFU/mL). This research indicates the possibility of employing this biosensing system for the detection of pathogens affecting human health. As a last example, alginate is a well-known natural anionic polymer employed in biomedical applications due to its high biocompatibility, hydrophilicity, nontoxicity, and affordability,^34,55^ which make it an ideal candidate for the development of wearable biosensors.^55^ Su et al.^72^ developed a conductive hydrogel where CS and sodium alginate (SA) formed the first physically cross-linking network, while the second one was formed though the ionic cross-linking between Ca^2+^ and SA or CS, which greatly enhanced the ionic conductivity of the hydrogel (9.80 S/m). Due to physical interactions, the hydrogels were able to reshape after being compressed (highest breaking strength was 281.51 kPa), twisted, and stretched and adapt to complex external conditions. This conductive hydrogel could adhere to human skin and monitor different motions, proving its potential applications in wearable flexible biosensors.^72^

**Figure 3:**
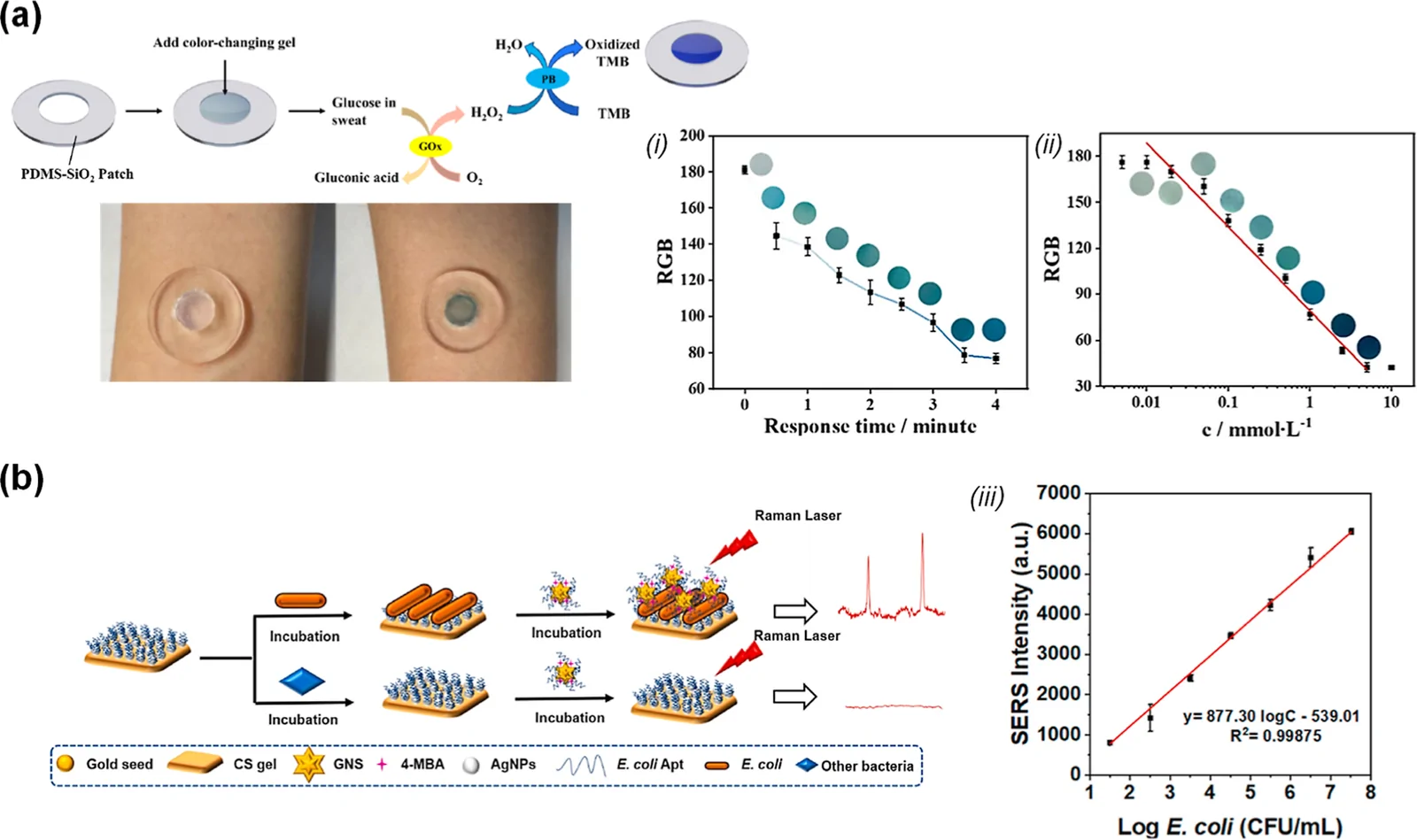
Natural polymer-based hydrogels for biosensing applications. (a) Schematic diagram of the device assembly process and mechanism for a wearable flexible agarose hydrogel patch sensing system. (i) Change in color-changing RGB with time at a concentration of 1 mmol/L of glucose. (ii) Fitted curve of the relationship between different concentrations of glucose and RGB. Reproduced with permission from ref 66 Copyright 2025 American Chemical Society. (b) Schematic diagram of SERS detection of *E. coli* with a capture probe Apt-AgNPs-CS gel. (iii) Detection performance of the SERS aptasensor. Raman spectra were recorded at different *E. coli* concentrations, and the corresponding SERS intensity at 1588 cm^–1^ was plotted against *E. coli* concentration, showing the linear relationship used for quantitative analysis. Reproduced with permission from ref 70 Copyright 2022 Elsevier.

### 2.2 Synthetic Polymer-Based Hydrogels

Synthetic polymer-based hydrogels are 3D networks of covalently or ionically cross-linked hydrophilic homopolymers or copolymers such as poly­(hydroxyethyl methacrylate) (PHEMA), polyacrylamide (PAM), polyethylene glycol (PEG), poly­(acrylic acid (AA)) (PAA), and poly­(vinyl alcohol) (PVA), among others.^73^ Compared to natural polymers, synthetic polymers usually have well-defined structures that can be modified to obtain the desired mechanical properties and functionality, although some of them present cytotoxic properties.^74^ They usually possess a higher water absorption capacity than natural hydrogels, which confers the hydrogel a degree of elasticity closer to that of the natural tissue.^73^ Therefore, they have gained special interest in the development of smart hydrogels for biomedical purposes.^34^

PEG is a synthetic polymer suitable for different biomedical applications, as it possesses excellent biocompatibility, solubility in water, and nonfouling properties.^75^ In recent studies, PEG and its derivatives have been employed in the development of different electrochemical, optical, and mass biosensors.^76^ For example, De Masi et al.^77^ designed PEG-based hydrogel microparticles for biosensing. Interestingly, by copolymerizing polyethylene glycol diacrylate (PEGDA) with N,N’-(1,2-dihydroxyethylene)-bis­(acrylamide) (DHEBA), a cleavable cross-linker, researchers introduced new pores and reactive groups while maintaining the mechanical integrity of the hydrogel. This structural modification ensured full accessibility of large proteins to the immobilized probes. The optimized microparticles were functionalized with antibodies capable of binding human IgG and demonstrated efficient target capture in the picomolar range from 0.1 to 1000 pM, indicating a picogram-level detection limit (Figure 4a). Consequently, the resulting porous microparticles exhibited the ability to act as molecular filters based on the biomolecule size. This approach enhanced the transport of the biomacromolecules inside the hydrogel and their interaction with the immobilized antibodies, thereby significantly improving the sensitivity of immunoassays based on porous microparticles. Poly­(EG)-grafted poly­(AA) (VPEG-*g*-pAAc) hydrogels have exhibited reversible swelling and tunable pore size that enabled controlled analyte transport for biosensing and bioanalytical applications.^78^

**Figure 4:**
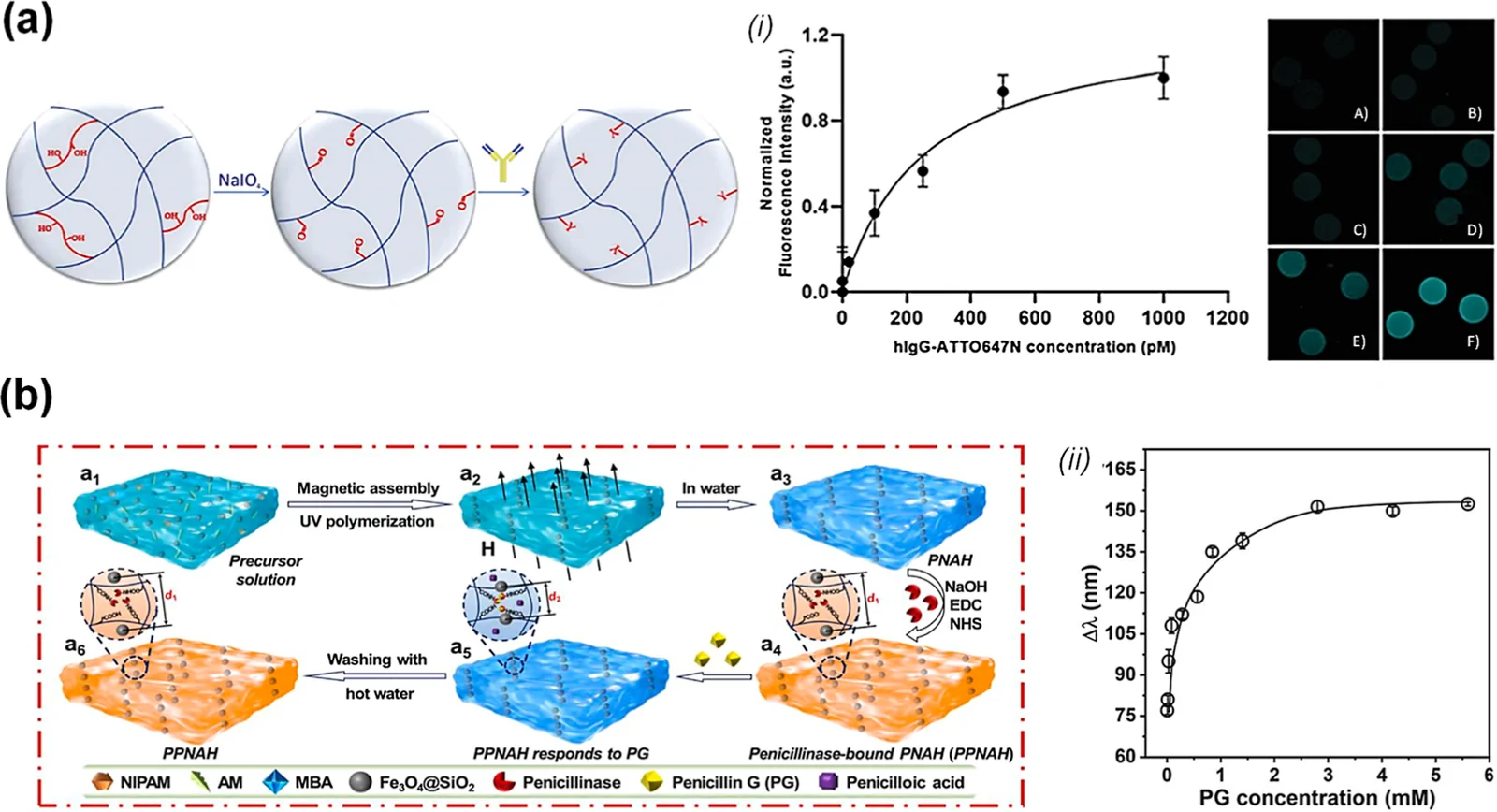
Synthetic polymer-based hydrogels for biosensing applications. (a) Schematic illustration of the hydrogel microparticle composed of a PEG diacrylate/DHEBA copolymer network functionalized with 0.1 pM anti-hIgG antibody that cleaves upon target binding, enabling fluorescence detection of hIgG-ATTO647N. (i) Normalized fluorescence signal intensity of hIgG-ATTO647N captured by functionalized hydrogel microparticles with 0.1 pmolpart–1 of ant*i*-hIgG antibody, and confocal fluorescence images of functionalized microparticles detecting atto647N–hIgG at different concentrations: 1, 10, 100, 250, 500, and 1000 pM (A–F). Reproduced with permission from ref 77 Copyright 2022 The Royal Society of Chemistry. (b) Schematic illustration of the fabrication (a1→ a4), penicillin G (PG) recognition (a4→a5), and regeneration (a5→a6) of the PPNAH biosensor. Graph (ii) represents the effect of the PG concentration on the response performance of the PNAH sensor to PG aqueous solution containing 0.1 M NaCl. Reproduced with permission from ref 84 Copyright 2024 Elsevier.

PVA is also a versatile synthetic polymer known for its excellent mechanical properties, superior biocompatibility, high water solubility, biodegradability, responsiveness to external stimuli, and ability to form stable networks through various cross-linking approaches.^79^ PVA-based hydrogels exhibit these advantageous characteristics, making them promising polymeric materials for a wide range of biomedical applications, including biosensing.^79–81^ For instance, Han et al.^81^ developed a highly stretchable, sensitive (Gauge factor (GF) of 2.94 over a tensile strain range of 0%–200%) and durable flexible strain sensor using a PVA-based hydrogel. The researchers created a hydrogel by combining PVA with sodium carboxymethyl cellulose (CMC), acrylamide (AM), AA (AAc), and zinc chloride, followed by addition of EG to improve moisture retention and freeze resistance. The final hydrogel showed excellent mechanical properties; it remained stable at temperatures as low as −41.6 °C and retained most part of its weight (83.7%) after 7 days, showing strong resistance to drying. In addition, it responded to temperature, sweat, and pH changes, making it suitable for multifunctional wearable sensors.

PAM is another synthetic polymer derived from acrylamide monomer commonly used in the polyacrylamide gel electrophoresis (PAGE) technique for the separation and analysis of biomolecules based on size, conformation, and net charge. However, PAM hydrogels are gaining interest among the biosensing field.^82,83^ Wang et al.^84^ developed a responsive photonic hydrogel (RPH)-based optical biosensor for sensitive and colorimetric detection of a typical β-lactam antibiotic PG in water (Figure 4b).

The biosensing system was formed by embedding Fe_3_O_4_@SiO_2_ colloidal photonic crystals (CPCs) into a poly­(*N*-isopropylacrylamide-*co*-acrylamide) smart hydrogel (PNAH). Subsequently, the PNAH hydrogel was functionalized with penicillinase as the bioreceptor, via amidation reactions between –NH_2_ in penicillinase and –COOH in the hydrolyzed PNAH, constructing the penicillinase-modified poly­(NIPAM-*co*-AM) hydrogel (PPNAH) biosensor. Upon immersing the functionalized hydrogel in a PG solution, the entrapped penicillinase could effectively catalyze PG hydrolysis into penicilloic acid, which increased the –COOH concentration in the hydrogel microenvironment, causing a significant reduction in pH and negative charges. This resulted in the spacing of the Fe_3_O_4_@SiO_2_ CPCs and the consequent change in the diffraction and structural color of the hydrogel from orange to blue. To assess the response performances of PPNAHs to PG, the fabricated PPNAH was soaked in PG solutions with various concentrations, and the relationship between the diffraction wavelength (Δ*λ*) and PG concentration was evaluated. The biosensor was able to detect PG concentrations ranging from 2.8 μM (LOD) to 2.8 mM, with a rapid initial response within the first 10 min. In addition, the PG-responded PPNAH could be conveniently regenerated through at least 50 cycles of alternative washing using hot/cold water without compromising its detection performance. PAA is a synthetic polymer that forms hydrogels with good elastic properties, highly sensitive to changes in pH due to their abundant carboxyl groups, and with self-healing and adhesive properties.^85^ For instance, Hussain et al.^86^ generated an optical photonic biosensor for the detection of microcystin (MC). Researchers fabricated a biosensor platform based on a interpenetrating polymeric network (IPN) within solid-state cholesteric liquid crystals (CLC_solid_), which are a class of photonic materials and have emerged as a promising foundation for robust sensors.^87^ Due to their capability to exhibit color changes caused by specific stimuli, CLC_solid_ have gained attention in the field of biosensors.^86^ The IPN was a PAA network, to which an amino-terminated short-chain MC aptamer was covalently attached, forming an innovative IPN_aptamer_–CLC_solid_ biosensor chip. After testing the fabricated chip in the presence of various concentrations of MC, the device was able to accurately measure various concentrations of MC (50, 100, 130 nM) dissolved in tap water.

Apart from those, PHEMA has also attracted a growing interest in the biosensing field due to its biocompatibility, optical transparency, hydrophilicity, and nondegradability.^88–90^ For instance, Li et al.^90^ fabricated a flexible and noninvasive electrochemical glucose biosensor based on a PHEMA hydrogel (LOD of 9.99 μM and a wide linear range of glucose concentrations of 0–0.2 mM and 0.2–4 mM). To enhance conductivity, the biosensor was doped with copper nanoparticles (CuNPs). The addition of CuNPs not only increased the conductivity of the hydrogel but also showed excellent electrocatalytic activity for the oxidation of glucose. The results showed superior recyclable adhesion on skin, as well as excellent self-healing capability of electrochemical performance for blood glucose monitoring.^90^ Rungsima et al.^91^ developed a pH-sensitive sensor capable of detecting acid–base solutions (pH range from 3.9 to 8) by mixing an anion type indicator dye, Bromothymol blue (HBTB), into the polymerization reaction between 2-hydroxyethyl methacrylate (HEMA) and diallyldimethylammonium (DADMAC) monomers. As the DADMAC content increased, the hydrogel became more acidic, which caused a noticeable color change from blue to yellow due to the presence of HBTB (a pH-sensitive dye). The hydrogel’s sensitivity to acidic and basic solutions, along with its color responsiveness, suggested the potential applications as a biosensor, such as detecting urinary overflow in elderly individuals. Moreover, photosensitive hydrogels have also attracted attention for biosensing.^92,93^ For instance, Chen et al.^94^ developed a stimuli-responsive hydrogel for the semiquantitative detection of tear glucose. A type of 3D photonic crystal sensor was created by embedding two dimensionally (2D) assembled polystyrene (PS) colloids into a 4-boronobenzaldehyde (4-BBA)-modified PVA hydrogel. These PS colloids arranged into a structure that reflects specific colors of light. When hydrogels around the CCA swell or shrink in response to changes like humidity, pH, or metal ions, the reflected color changed, allowing the sensor to detect these stimuli. According to results, the interaction between glucose molecules (in a physiological concentration range) and the hydrogel’s network altered the hydrogels volume, particularly the spacing of the PS colloids relating to glucose combination, and thus, the Bragg diffraction of the hydrogel shifted. The sensor demonstrated a highly accurate, sensitive, and rapid response (<180 s) in detecting glucose concentration in tears from 0 to 20 mM, being a potential candidate for POC glucose monitoring.

### 2.3 Hybrid and Semisynthetic Polymer-Based Hydrogels

In the electrochemical biosensing research field, ionically conductive polymer-based hydrogels have emerged as the material of choice, attracting increasing attention from the scientific community.^16,95,96^ In this case, the polymer network makes the hydrogel an elastic solid, and the water molecules make the hydrogel an ionic conductor.^97^ Moreover, ionic conductive hydrogels can be prepared also by increasing the ionic conductivity of the aqueous phase using salts, ionic liquids (ILs), and deep eutectic solvents (DES).^41,98^ In this context, Pan et al.^99^ recently developed a wearable device based on an ionic conductive hydrogel that consisted of a sulfobetaine methacrylate (SBMA) and acrylamide (AM) copolymerized network doped with NaCl. The hydrogel showed high ionic conductivity (190 mS m^–1^) and high stretchability (2003% strain). According to their successful results, by combining the proposed hydrogel with wearable devices, these researchers could continuously monitor and compare the electrocardiogram signals between healthy and diabetic people.^99^

Recent research has also been focused on the development of both ionic and electronic conductive hydrogels.^100,101^ Electronic conductive polymer hydrogels are hybrid materials created by incorporating an electrically conductive pathway into the hydrogel polymer network during or after the gel formation.^65,102^ In this case, the cross-linked hydrogel network acts as the physical scaffolding, and the conductive material imparts electrical or ionic conductivity and functional versatility.^22^ The high water content creates conditions for the directional movement of electrons, thus giving conductivity to traditional hydrogels.^103^ These conductive hydrogels based on nonconducting polymers allow a wider range of options in the selection of the natural or synthetic polymer. Moreover, the functionalization of the hydrogel with conducting polymers (CPs) frequently leads to a nontransparent hydrogel, which limits the direct visualization.^103^ The potential of tuning both the mechanical strength and the conductivity values of the hydrogel offers a wide variety of applications, such as hydrogel-based strain sensors.^102^ Nevertheless, although many well-developed hybrid hydrogels exist, few of them exhibit all the requirements to act as wearable, flexible, injectable, and conductive hydrogel-based biosensors.^104,105^ These hybrid hydrogels can be prepared through different assembly techniques, by incorporating intrinsically metallic nanomaterials such as metal microwires or nanoparticles, quantum dots (QDs), or carbon-based materials such as carbon nanotubes (CNTs) into the polymer hydrogel to endow it with electronic conductivity.^22,41^ Compared to ionic conductive polymer-based hydrogels that may suffer from diffusion and leakage of ions to the surrounding environment, electrical conductive polymer-based hydrogels exhibit a better biocompatibility with biological tissues.^100^

Despite the advantages of functionalizing hydrogels with conductive nanomaterials, they still may have some limitations that impose barriers in their application on wearable biosensors. Challenges may arise from the poor biocompatibility between hydrogels and traditional metal nanoparticles, as well as the inherent biotoxicity of these conducting fillers.^103^ Recent studies have focused on addressing these limitations. Particularly, liquid metal (LM) has emerged as an innovative conductive filler in the development of wearable soft electronic devices.^106^ LM can not only give the hydrogel high conductivity and biocompatibility but also solve the internal stress caused by the mismatch of mechanical properties at the interface between the hydrogel network and traditional rigid conductive fillers, such as CNTs, conferring higher stretchability.^106,107^ For instance, Sun et al.^108^ connected LM nanoparticles by CNTs, forming LM/CNTs composites, which were later added to a double-network hydrogel formed by PAM and SA. This approach endowed the hydrogel with high electrical conductivity (94 S m^–1^), excellent optical transparency (93%), high adhesion strength (20 kPa), ultrahigh stretchability (2200%), and rapid self-healing capability (self-healing time in 20 min). These properties enabled the recording of high-quality heartbeat signals and real-time visual observation of cardiac bleeding, contributing to the successful performance of the first minimally invasive cesarean procedure on Chinese sturgeon. In addition, graphene and graphene derivatives are commonly used materials in the development of biosensors due to their exceptional electrical conductivity.^109^ However, the application of conventionally synthesized reduced graphene oxide (rGO) as a biomaterial is severely restricted by its tendency to aggregate.^110^ Jiang et al.^111^ developed electroactive composite hydrogel sensors composed of PAA and rGO. Researchers employed an interesting approach in which rGO was obtained through the partial conversion of GO to graphene through dopamine oxidation, inspired by polydopamine (PDA) polymerization.^112^ However, the composite hydrogels possessed relatively low conductivity due to the inadequate dispersion of the PDA-rGO nanosheets. Dou et al.^110^ developed a hydrogel utilizing heparin-polydopamine (Hep-PDA)-reduced rGO nanosheets (Hep-PDA-rGO), which were incorporated into a PAM hydrogel matrix to create conductive hydrogels (Hep-PDA-rGO-PAM) with a high electrical conductivity (up to 3.63 S m^–1^) to be used as epidermal sensors. This successful approach enhanced the biocompatibility (HEF1 fibroblasts showed good viability and growth on the hydrogel) and stability of rGO in the network, as well as confer the conductive hydrogel with desirable properties such as antioxidant activity, antibacterial activity, and excellent sensing performance.^110^

Semisynthetic polymer-based hydrogels are formed from the combination of natural polymers with compatible synthetic polymers through chemical or physical cross-linking. This approach allows the incorporation of reinforcing components, effectively enhancing material performance while mitigating the individual limitations of each polymer type to obtain hydrogels with multifunctional properties.^113,114^ For example, Karuppaiah et al.^45^ developed an electrochemical salivary cortisol biosensor that involved a hydrogel-nanocomposite layer comprising gold nanocubes (AuNCs) accompanied by a cortisol-specific aptamer to monitor diverse biomarkers. The polymer hydrogel, made of chitosan (CHI) and hydroxyethyl cellulose (HEC), helped to reduce the interactions between the electrode and nonspecific biological macromolecules in the biofluid. AuNCs were used as nanofillers to enhance the conductivity and direct encapsulation of cortisol-specific aptamers.^45^ According to the results, the biosensor enabled a wide detection range (0.1–50 ng/mL) with an LOD of 0.1 ng/mL. A recent study developed a double network hydrogel strain sensor formed by SA, tannic acid, acrylamide (AM), and 2-acrylamide-2-methylpropanesulfonic acid (AMPS), integrated with ILs and multiwalled CNTs.^115^ The IL effectively allowed the dispersion of the multiwalled CNTs, enhancing the conductivity (5.37 × 10^–2^ S/m), high sensitivity (GF of 0.12 for 40%–100% strain and 0.24 for 100%–250% strain), self-adhesive properties (0.039 MPa), and biocompatibility of the hydrogel. Therefore, the integration of natural polymers and advanced materials highlights the potential of biomacromolecules in creating innovative hydrogel-based sensors for biomedical applications.^115^

### 2.4 CP-Based Hydrogels

CPs are organic macromolecules that possess intrinsic and exceptional electrical conductivity while retaining the properties of common polymers, such as soft mechanical properties, high flexibility, and low mass density.^116–118^ Due to these advantages, CPs have been integrated in the biomedical field and widely used for biosensing.^119,120^ Hydrogels based on CPs (CPHs) (e.g., polypyrrole, polyaniline, and poly­(3,4-ethylenedioxythiophene)) were first synthesized in the 1990s by Guiseppi-Elie et al.^121^ CPHs can be fabricated either from pure CPs or from the incorporation of CPs into a supporting hydrogel.^122^ Depending on the design methods, CPHs can be tuned in terms of electrical properties and mechanical properties.^123^ However, the mechanical properties and biodegradability of CPs are not ideal for clinical applications.^122^ This issue can be overcome by the combination with a supporting polymer that helps to improve the hydrophilicity and mechanical strength of the hydrogel network.^124^ The creation of these “hybrid” CPHs can be achieved either by the polymerization of the CPs within a preestablished hydrogel network or direct mixing of the CPs and hydrogel monomers with simultaneous polymerization.^124^ Polyaniline (PANI) has garnered significant interest among conductive polymers due to its high polymerization efficiency and conductivity.^125^ The presence of amine groups (–NH_2_) within PANI facilitates its chemical modification to be used for bioreceptors encapsulation, making it very desirable for the fabrication of biosensors.^126^ Yang et al.^126^ developed a PANI/Phytic acid (PA) CPH by one-step electrochemical copolymerization (Figure 5a). In a second step, carboxyl-functionalized DNA probes were covalently attached to the amino groups of the hydrogel using *N*-(3-(dimethylamino)­propyl)-*N*′-ethylcarbodiimide (EDC) and *N*-hydroxysuccinimide (NHS) as coupling reagents. This electrochemical biosensor demonstrated excellent selectivity for MiRNA24 and a wide linear range of detection (1.0 fM–1.0 pM), which plays important roles in cancer cell proliferation and differentiation.^126^ Poly­(3,4-ethylenedioxythiophene) (PEDOT):polystyrenesulfonate (PSS) conductive hydrogels have also been used in applications related to conductive hydrogels.^127–129^ In particular, PEDOT:PSS hydrogels can be easily fused with ions or monomers to polymerize interpenetrating porous and soft networks.^130^ However, traditional design methods of PEDOT:PSS hydrogels led to bulk aggregates of PEDOT chains, which resulted in undesirable mechanical properties such as low stretchability (20%).^131^ Chong et al.^16^ reported a novel template-direct assembly method and conduction mechanism to obtain a highly conductive, soft, adhesive, and tough PEDOT:PSS/AA hydrogel network for bioelectronic applications. The use of a template network with a high binding affinity with the PSS shell suppressed the aggregation of PEDOT chains, forming a homogeneous conductive network. As another example, Xu et al.^132^ developed an electrochemical biosensor based on a PEDOT:PSS conductive hydrogel containing Prussian blue nanoparticles (PBNPs) for noninvasive, continuous monitoring of glucose in the body (Figure 5b). It is noteworthy to mention that despite the wide range of commercially available glucose monitoring technologies, no noninvasive method currently exits for the continuous measurement of glucose in interstitial fluid (ISF).^132^ The only technology that exists for noninvasive ISF glucose uses reverse iontophoresis (RI) to extract ISF through the skin via electroosmosis.^133^ With this method, ion flow occurs due to a small electric field applied across the skin, and glucose goes from the skin (net negatively charged) toward the cathode on the skin surface. To improve the contact between the electrode and skin, a thin hydrogel was used, providing storage for glucose reaction with GOx. Therefore, it is critical to have a highly conductive hydrogel with strong electrocatalysis sensing properties. PBNPs demonstrated outstanding electrocatalytic activity and PEDOT:PSS showed higher conductivity and extensive electron transport. The glucose biosensor could monitor the ISF glucose on two volunteers’ skin surface, showing excellent electrochemical properties compared to commercial glucometers, such as an LOD of 0.85 μM for glucose detection and a high sensitivity up to 340.1 μA·mM^–1^·cm^–2^. Polypyrrole (PPy) is another commonly used CP, which is obtained from the oxidation of pyrrole or substituted pyrrole monomers.^134^ However, pyrrole and polypyrrole are insoluble in water, which limits the preparation of conductive PPy-incorporated hydrogels.^125^ In this case, PPy hydrogels are prepared by the swelling method. For instance, Han et al.^135^ developed a conductive hydrogel of silk/PPy by immersing the silk hydrogel in pyrrole monomers to absorb the pyrrole molecules into it. Then, the pyrrole molecules were chemically polymerized within the silk hydrogel by immersing it into an FeCl_3_ solution. The resulting silk/PPy hydrogel showed a good performance as a strain sensor. Wang et al.^68^ synthesized a water-soluble polypyrrole that was used to prepare a series of conductive hydrogels by mixing it with chitosan and cucurbit[7]­uril. The resulting hydrogels showed superior conformability, stability (stable over 1000 sensing cycles), biocompatibility (98.25% of cell viability), and conductive properties (electrical conductivity up to 0.534 S m^–1^) and were able to detect not only various body movements but also physiological signals.^68^ Hybrid conductive polymer hydrogels were developed by Liu et al.^136^ They fabricated a salivary glucose biosensor based on core–shell PPy hydrogel/carbon nanotubes (CNTs) on flexible carbon papers (CP). The prepared CNTs and the pyrrole monomer solution were drop-casted onto the pretreated CP electrode, and polymerization was carried out by drop-casting APS solution. For enzyme immobilization, GOx solution was drop-casted onto the PP_Y_/CNTs/CP, followed by the drying process under vacuum for 3 h. To evaluate the application of the GOx/PP_Y_/CNTs/CP biosensor in salivary glucose monitoring, the glucose concentrations of commercially available artificial saliva were determined. The device demonstrated a high glucose-responsive capability, with a linear-range detection of 100–700 μM and high sensitivity (119.74 μA/mM·cm^2^).

**Figure 5:**
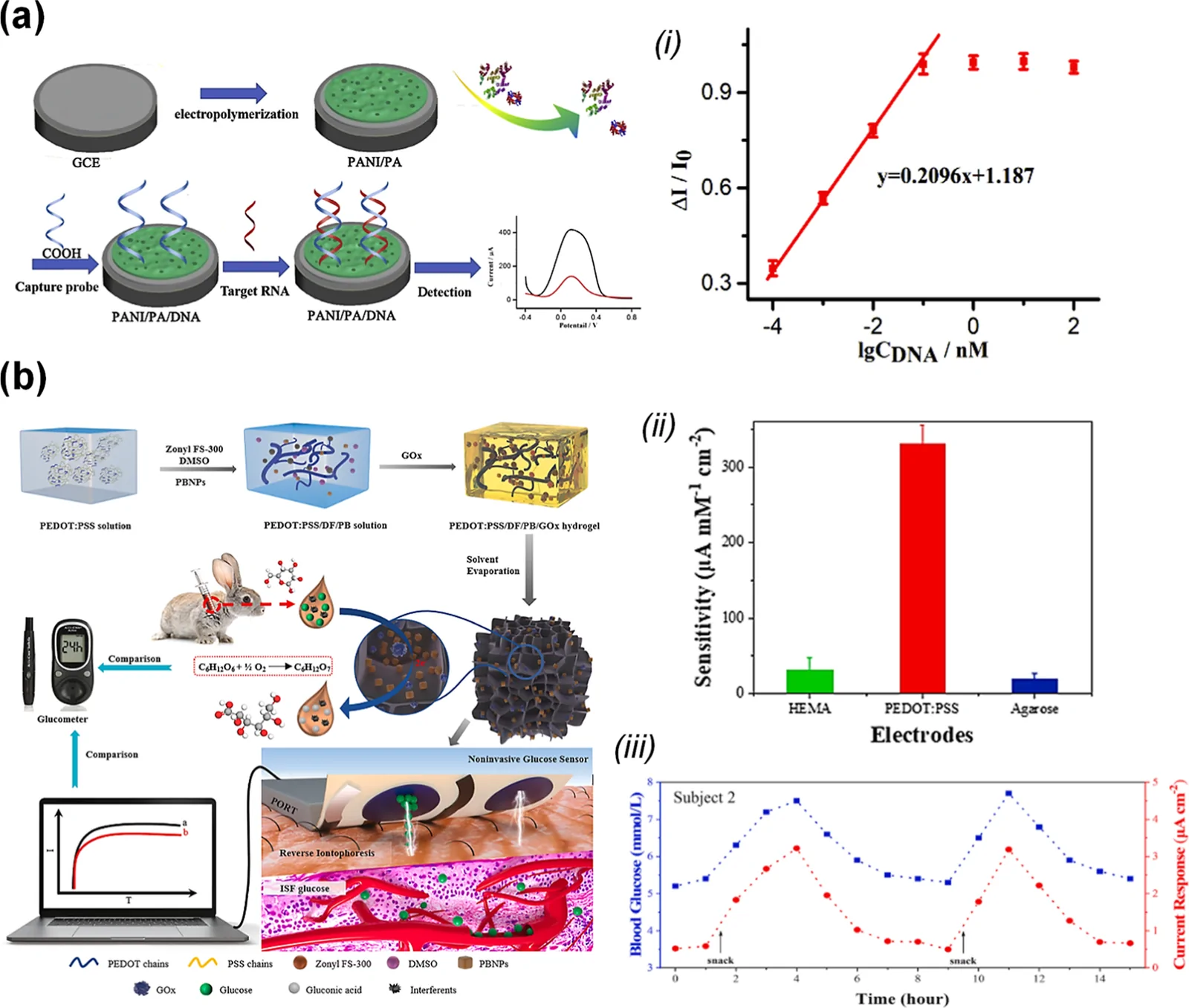
CP-based hydrogels for biosensing applications. (a) Schematic illustration of the structure of the PANI/PA antifouling interface and its application for the miRNA24 sensor. (i) Calibration curve of the biosensor with different concentrations of RNA24 in PBS pH 5.7. Reproduced with permission from ref 126 Copyright 2022 Elsevier. (b) Schematic demonstration of the preparation of the PEDOT:PSS/PB/GOx biosensor for the glucose detection in serum and the noninvasive ISF glucose detection on human skin based on the RI, along with comparison to the glucose measurement by a commercial glucometer. (ii) Glucose detection sensitivity with different hydrogel sensor chips, including the HEMA/PB/GOx, PEDOT:PSS/DF/PB GOx, and agarose/PB/GOx GCE. (iii) Noninvasive ISF glucose current response measured every hour versus the blood glucose measured 15 min before the ISF extraction. Reproduced with permission from ref 132 Copyright 2022 Elsevier.

#### 2.4.1 Intrinsic versus Hybrid Approaches in Conductive Polymer Hydrogels

As conductive hydrogels continue to gain importance in electrochemical biosensing, it is essential to distinguish between intrinsically conductive polymer hydrogels and nanofiller-based hybrid conductive hydrogels.^137^ While both material classes are designed to facilitate charge transport within hydrated networks, they rely on different conductivity mechanisms and exhibit distinct electrochemical, mechanical, and biological characteristics. A critical differentiation between these two material classes is therefore necessary for the rational design of electrochemical biosensors. Intrinsically conductive polymer hydrogels derive their electrical conductivity from the continuous conjugated backbone of CPs such as PANI, PPy, and PEDOT. In these systems, charge transport primarily occurs through electronic conduction via delocalized π-electrons along the polymer chains and charge hopping between adjacent conductive domains. Consequently, the conductive component itself actively participates in electron transfer processes, often serving simultaneously as the sensing interface, transducer, and hydrogel matrix. This architecture generally results in efficient electron transfer kinetics, high signal amplification, and strong coupling between immobilized bioreceptors and the electrode surface.^138,139^ In contrast, nanofiller-based hybrid conductive hydrogels achieve conductivity through the incorporation of conductive fillers into an otherwise insulating polymer network. These fillers may include CNTs, graphene derivatives, metal NPs, liquid metals, QDs, or conductive nanocomposites. Electrical transport in such materials occurs through the formation of percolation networks, where electrons move across interconnected conductive fillers embedded within the hydrated polymer matrix. The hydrogel primarily provides mechanical support, water retention, and biocompatibility, while the conductive nanofillers act as the main electron transport pathways. Therefore, conductivity in hybrid hydrogels strongly depends on filler concentration, dispersion homogeneity, interparticle connectivity, and long-term structural stability.^140,141^

From an application perspective, intrinsically conductive polymer hydrogels are particularly suitable for electrochemical biosensors where rapid electron transfer and high analytical sensitivity are the primary requirements, such as enzymatic glucose sensing, nucleic acid detection, and electrochemical affinity biosensors. On the other hand, nanofiller-based hybrid conductive hydrogels are especially attractive for wearable and implantable biosensors that require simultaneous conductivity, mechanical compliance, self-healing ability, transparency, and tissue adhesion. For example, LM- and graphene-based hydrogel composites can tolerate large deformations while maintaining stable electrical signals, making them highly suitable for epidermal sensors and continuous physiological monitoring systems. Many modern conductive hydrogels combine CPs with conductive nanofillers to exploit the advantages of both the approaches. Such hierarchical conductive architectures can simultaneously provide efficient electron transport, enhanced mechanical properties, and multifunctional sensing capabilities. Future research will likely focus on optimizing these hybridized systems while minimizing interfacial resistance, improving long-term biocompatibility, and achieving stable conductivity under dynamic physiological conditions.

### 2.5 Biodegradable Polymer-Based Hydrogels

Biodegradable polymer-based hydrogels can be derived from natural, synthetic, or hybrid polymer systems and are an important class of materials for next-generation biosensing. They combine the highly hydrated three-dimensional architecture characteristic of conventional hydrogels with the ability to undergo controlled degradation under physiological or environmental conditions, generating nontoxic byproducts that can be metabolized or safely eliminated from the body.^142^ The degradation of polymeric hydrogels can proceed through hydrolytic, enzymatic, oxidative, or stimulus-triggered mechanisms, depending on the chemical structure of the polymer network and the surrounding environment.^143^ Natural polymers such as collagen, gelatin, alginate, chitosan, hyaluronic acid, and dextran are inherently biodegradable and typically degrade through enzyme-mediated processes. In contrast, synthetic biodegradable polymers, including poly­(lactic acid) (PLA), poly­(glycolic acid) (PGA), poly­(lactic-*co*-glycolic acid) (PLGA), polycaprolactone (PCL), and degradable polyethylene glycol (PEG)-based derivatives, primarily undergo hydrolytic degradation and offer greater control over degradation kinetics and mechanical performance.^144–146^

The controlled degradation of hydrogel matrices offers several advantages for biosensing applications. In implantable devices, biodegradable hydrogels can reduce long-term foreign-body responses and eliminate the need for surgical retrieval after use.^147^ Furthermore, degradation can be engineered to regulate sensor lifetime, analyte transport, and the release of therapeutic or signaling molecules, enabling the development of transient and multifunctional sensing platforms.^144–146^ These characteristics have generated considerable interest in biodegradable hydrogel-based sensors for monitoring glucose, metabolites, inflammatory biomarkers, and other clinically relevant analytes.^148^ Recent advances have expanded the functionality of biodegradable hydrogels through their integration with conductive polymers, inorganic nanomaterials, and stimuli-responsive components, enabling the simultaneous achievement of biodegradability, signal transduction, and molecular recognition.^149^ These multifunctional systems have shown considerable potential for wearable and implantable biosensors, as well as emerging bioresorbable electronic devices.^150^ Nevertheless, several challenges continue to hinder their clinical translation.^151^ Precise control over degradation kinetics remains critical, as premature degradation may compromise signal stability, sensitivity, and device lifetime, whereas excessively slow degradation may reduce the advantages of bioresorbability. In addition, degradation-induced changes in porosity, swelling behavior, mechanical properties, and analyte diffusion can affect sensing accuracy and long-term device performance.^151^ A further challenge arises from the incorporation of conductive polymers and inorganic nanomaterials within biodegradable hydrogel matrices, since many of these functional components are not inherently biodegradable.^152^ Consequently, while the polymer network may undergo complete degradation, residual conductive or nanostructured materials may persist within the biological environment, raising concerns regarding long-term biocompatibility, clearance, and environmental sustainability. Addressing these limitations will require the development of fully biodegradable conductive materials and multifunctional hydrogel systems with predictable degradation profiles and stable sensing performance throughout their operational lifetime.

## 3 Bioreceptor Integration and Functionalization Strategies for Enhanced Chemical Biosensing

Despite the attractive properties of hydrogels for chemical biosensing, several limitations still hinder their performance, including insufficient molecular recognition capabilities, poor mechanical robustness, nonspecific interactions, and restricted analyte transport within the polymer network. To address these challenges, a variety of bioreceptor integration and functionalization strategies were developed. These approaches aim to improve hydrogel stability, selectivity, sensitivity, and signal transduction by tailoring the material surface, incorporating biological recognition elements, and engineering the hydrogel architecture at the microscale. The following sections discuss recent advances in surface modification, biofunctionalization, and micropatterning strategies for enhancing the hydrogel-based chemical biosensors.

### 3.1 Surface Modification

Although polymer-based hydrogels offer numerous advantages, they also face several common challenges, such as nonspecific protein binding, undesired bacterial adhesion, and surface deformation during swelling in contact with biological fluids.^153^ The mechanical properties of hydrogels are influenced by factors such as the polymer structure, molecular weight, network topology, and cross-linking type and density. However, most hydrogels are synthesized via random polymerization, which often leads to inhomogeneous networks and mechanical defects, limiting their use for biomedical applications.^154^

Particularly, for applications like flexible strain biosensors, high toughness and fatigue resistance are essential.^50^ To address these issues and enhance the mechanical performance, surface modification techniques have been commonly employed.^155–158^ As previously explained, hydrogels can be cross-linked either physically or chemically. Compared with physical cross-linking, chemically cross-linked hydrogel networks have better mechanical properties and stability.^42^ Among the different chemically cross-linking mechanisms, “click” chemistry has gained significant attention in the field of hydrogel synthesis for biomedical applications, owing to its rapid reaction rate, simple conditions, and high selectivity.^158,159^ “Click” chemistry is not a single specific reaction, but a general term for many reactions with the same characteristics (CuAAC reaction, SPAAC reaction, Thiol-ene reaction, Diels–Alder reaction, nucleic acid “click” chemistry, etc.).^158^ “Click” chemistry offers a versatile approach for cross-linking hydrogel networks, enabling the implementations of various strategies to customize their physical and chemical characteristics.^158^ As an example, Thongrom et al.^160^ developed a biosensing platform utilizing thiol-click chemistry to fabricate polyglycerol (dPG) hydrogels. These hydrogels were designed to encapsulate streptavidin in situ, serving as a target for biotin detection. They employed thiol-Michael and thiol-ene additions to cross-link a previously functionalized dPG (with acrylate, allyl, or acrylamide groups) with dithiolated polyethylene glycol (PEG), creating a biocompatible 3D network. The thiol-click chemistry was highlighted for its high yield, ease of preparation, high specificity, and lack of byproducts, making it a suitable method for constructing hydrogels with precise control over their properties.

### 3.2 Biofunctionalization

Most of the previously mentioned polymers inherently lack the capability to specifically or strongly recognize target biomolecules, which means that unmodified hydrogels typically do not possess molecular recognition functionality.^161^ However, molecular recognition is essential for a wide range of biomedical applications, including protein delivery, regenerative medicine, and molecular biosensing.^162^ Therefore, biofunctionalization strategies have also expanded the biological responsiveness of hydrogel systems, and various biomolecules, including antibodies, enzymes, aptamers and cells, have been used as ligands to develop hydrogels with molecular recognition functionality.^45,163–165^

For instance, Wang et al.^166^ fabricated a novel optical glucose biosensor designed for real-time monitoring under physiological conditions. The sensor was based on a thin hydrogel film composed of partially oxidized dextran (PO-Dex), chitosan, and GOx, assembled using a layer-by-layer method.^167^ GOx acted as the bioreceptor, catalyzing the conversion of glucose to gluconic acid, which lowered the local pH and caused the hydrogel to swell. This swelling altered the optical properties of the film, producing a measurable shift in the Fabry–Perot interference correlated with glucose concentration. Results proved the rapid and reversible response, particularly within the clinically relevant range of 0–20 mM glucose, with high linearity and minimal interference from other biomolecules. Similarly, a self-healing hydrogel composed of carboxymethyl chitosan (CMCHI) and oxidized carboxymethyl cellulose (OCMC) was developed for glucose detection, with GOx serving as the key enzymatic recognition element (Figure 6a).^168^ GOx was encapsulated within the hydrogel matrix alongside gold nanoclusters (AuNCs) to form a fluorescent bioprobe. In the presence of glucose, GOx catalyzed the oxidation of glucose to produce hydrogen peroxide (H_2_O_2_), which in turn caused fluorescence quenching of the AuNCs. This enzyme-mediated signal transduction enabled the hydrogel to respond selectively and sensitively to glucose concentrations in the range of 100 μM– 5 mM, with a detection limit of 0.029 mM, covering the clinically relevant glucose range. The integration of GOx not only provided specific recognition of glucose but also ensured biocompatibility, making the biosensor suitable for in vitro and in vivo applications. Moreover, the self-healing property of the hydrogel, combined with the enzymatic activity of GOx, contributed to the durability and long-term stability of the biosensing system. In another work, Li et al.^169^ developed a difunctional hydrogel optical fiber for the simultaneous and continuous monitoring of pH and glucose levels. The biosensor was based on a poly­(acrylamide-*co*-ethylene glycol diacrylate) hydrogel core integrated with calcium alginate fibers to facilitate the optical signal transmission. Two sensing agents were incorporated, a fluorescein derivative for pH sensing and boronic acid-functionalized QDs for glucose detection. The fluorescein derivative was covalently immobilized within a segment of the hydrogel optical fiber, exhibiting a pH-dependent fluorescence response. The reversible protolytic equilibrium of fluorescein enabled rapid and repeatable pH sensing (3.4–9.0), while the glucose-sensitive region (0–20 mM) relied on the interaction between boronic acid and glucose to induce hydrogel swelling, which was transduced into fluorescence signal variations. Moreover, Chia et al.^170^ reported the first example of 3D porous calcium alginate hydrogel immuno-beads that were used to trap bioreceptors and preserve their bioactivity for cancer detection. These immuno-beads comprised two different monoclonal IgG capturing antibodies conjugated with two different fluorescent dyes to target two breast cancer biomarkers (HER2 and CA125 proteins). After incubating the beads with the serum sample for 20 min, the detection of these biomarkers through multiplex fluorescence quantification was successfully assessed, showing higher sensitivity (LODs of 0.004 ng/mL for serum HER2 and 0.005 U/mL for serum CA125) comparable to those obtained using other standard methods, such as the ELISA assay (LOD of 0.5–80 U/mL for CA125).^171^ In addition, alginate can be electrodeposited to locally functionalize electrodes. Through the application of a positive potential, water splitting is caused producing protons and lowering the pH. Then, CaCl_2_ dissociates, attempting to buffer the solution, and the free Ca^2+^ interacts as a cross-linker between alginic acid molecules, forming a gel. If this occurs in the presence of bioreceptors, they become trapped. This process has been used in electrochemical sensors to enzymatically measure glucose and lactate in blood and to measure ascorbic acid in sweat.^172,173^ Recently, Yamazaki et al.^174^ presented a novel, noncovalent method for functionalizing gelatin hydrogels with antibodies using a designed peptide tag (R9Dec2L3), which combined collagen-binding, hydrophobic, and electrostatic interactions to enhance antibody retention and activity. The tag significantly improved the adsorption and stability of an αCD3/28 agonistic antibody on the hydrogel surface, maintaining over 62% of its initial binding after 8 days in serum. Moreover, the agonistic activity of the anchored antibodies was preserved and improved, as evidenced by their ability to activate Jurkat/Luc-IL-2 reporter cells expressing luciferase through fluorescent signals derived from their interaction with the surface of the antibody–hydrogel complex. The approach achieved a high incorporation efficiency (22%) without chemical cross-linking, offering a simpler, more efficient alternative to traditional conjugation methods (Figure 6b).

**Figure 6:**
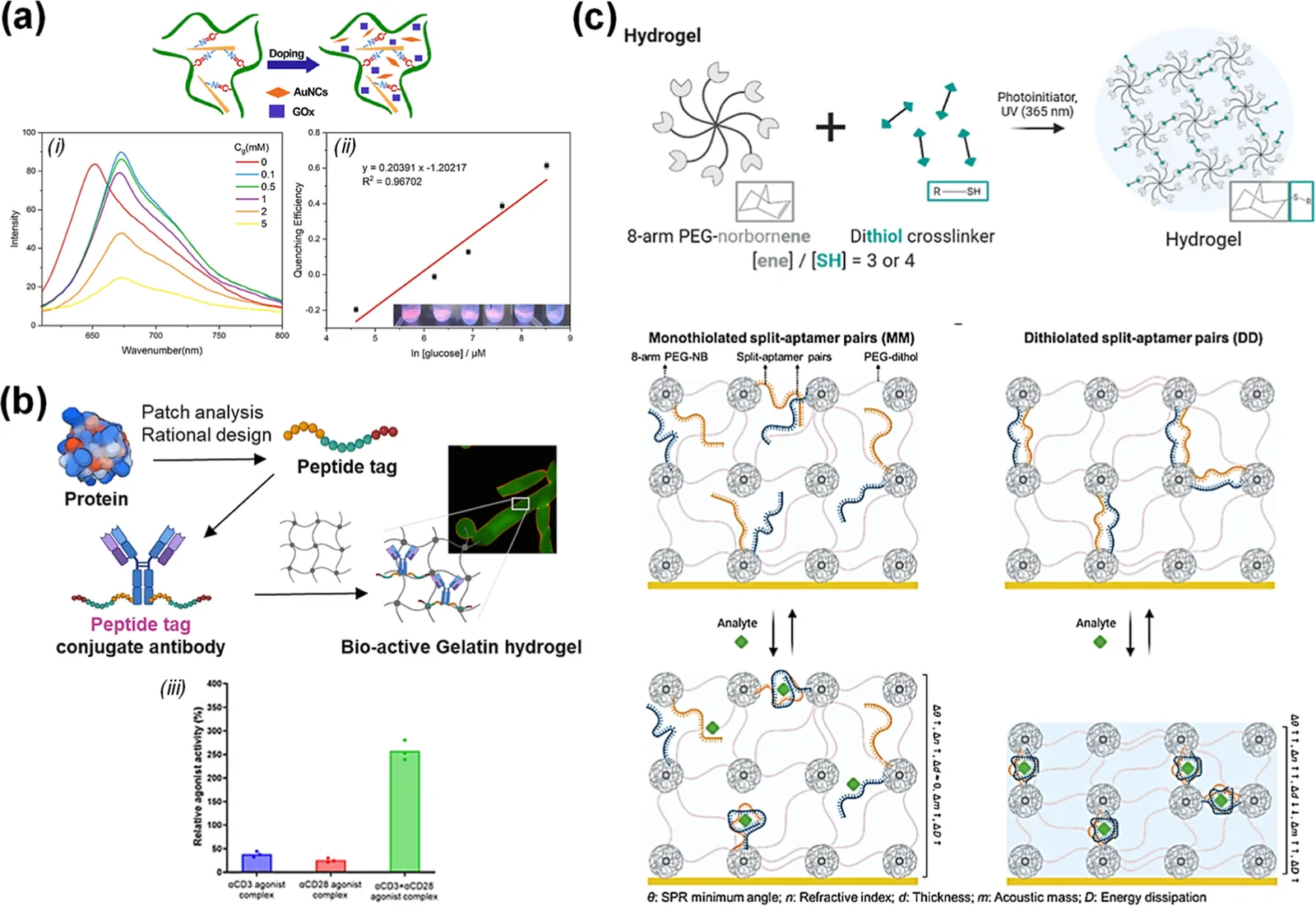
Functionalization strategies for enhanced chemical biosensing. (a) Schematic illustration of the AuNCs-CMCS/OCMC hydrogel. (i) Fluorescence intensity (FI) curves under different glucose concentrations. (ii) Calibration curve of the FL quenching efficiencies. Reproduced with permission from ref 168 Copyright 2021 Elsevier. (b) Design and fabrication of the bioactive gelatin hydrogel, including a fluorescence microscopy image of the gelatin hydrogel fabric with C-14 after being soaked in inactivated fetal bovine serum at 37 °C. Graph (iii) shows the highest relative luminescence intensity for the conjugated αCD3/28 antibody–hydrogel complex using protein tag. Reproduced with permission from ref 174 Copyright 2025 American Royal Society. (c) Schematic illustration of the structure of the hydrogel, composed of star-shaped PEG-NB and a dithiol cross-linker at a molar ratio of 3 or 4, leaving free norbornene groups available for covalent binding with free thiols on the gold surface (top). Representation of the proposed mechanism of dithiol and monothiol split–aptamer pairs upon analyte binding, leading to optical and acoustic changes in the hydrogel layer (bottom). Reproduced with permission from ref 178 Copyright 2025 American Chemical Society.

Apart from that, nucleic acid aptamers are a unique class of synthetic ligands that consist of a short single DNA or RNA strand with 20–60 nucleotides in length.^175^ In comparison to other recognition molecules, aptamers have received special interest for molecular biosensing applications, owing to their higher affinity, stability, and specificity in recognizing their target molecules, as well as their smaller size and low-cost production.^161^ Biosensors using aptamers as their bioreceptor element, defined as aptasensors, have been studied for various biomedical applications, such as for the detection of pathogens.^175^ Currently, aptamer-based hydrogel sensing platforms are gaining interest.^161,176,177^ For instance, Park et al.^178^ developed a molecularly responsive aptamer-functionalized hydrogel system for real-time and continuous plasmonic monitoring of complex biological processes, playing an essential role in the development of advanced biosensing platform for applications in biomedical diagnostics (Figure 6c). The polymer network was primarily based on PEG macromonomers functionalized with thiol and norbornene groups, which enabled the formation of a covalently cross-linked hydrogel through a thiol-ene click chemistry reaction. Remarkably, the hydrogel was functionalized with aptamers, acting not only as the recognition element but also as an improvement of the mechanical and functional properties of the network. The encapsulated aptamer enabled the hydrogel to respond dynamically to the presence of specific target molecules, such as vancomycin, by undergoing structural changes that could be detected through optical and plasmonic sensing. Particularly, increased aptamer concentrations in the hydrogel network revealed a lower LOD (160–250 nM of vancomycin), compared to other systems, as well as excellent reproducibility, wide detection range (up to 1 mM), and long-term stability up to 5 weeks in plasma-like conditions.

Cell-based hydrogels offer an interesting alternative to the previously mentioned bioreceptors, such as enzymes, antibodies, DNA, etc. Although these molecules are highly specific to several biological targets, living cells as bioreceptors possess enzymes and other molecules required for biosensing in their native environment.^179^ Hydrogel matrices can provide a stable 3D tissue-like environment for cell immobilization, growth, and proliferation. Moreover, hydrogels can also act as conductive electrodes for signal transduction through the incorporation of conductive materials or employing CP-based hydrogels.^3,128^ In this scenario, 3D bioprinting has emerged as a potential technique for micropatterning cells on different surfaces.^180^ This approach provides interesting properties for the development of cell-based biosensors, such as high throughput, controlled patterning, and rapid and precise deposition method.^181^ As an example, Choi et al.^182^ demonstrated the in vivo implantation of a cell-containing optical PEGDA-based hydrogel and its application in real-time cell-based toxicity sensing and light-controlled optogenetic production (10 pM) of an antidiabetic glucagon-like peptide-1 (GLP-1) in live mice. For the development of this biosensor, cells were incorporated into the precursor solution before UV-induced cross-linking, ensuring they were uniformly distributed within the hydrogel structure. The light-guiding capability of the hydrogels was enabled by their optical transparency and refractive index properties, which allowed the delivery of light to encapsulated cells for stimulation, as well as the real-time detection of optical signals from the cells for sensing purposes and implantable biodevices.

### 3.3 Hydrogels with Micropatterned Structures

Micropatterning hydrogels in a process similar to photolithography can be used to control and provide a 3D, high-surface-area environment for biomolecule and single-cell immobilization with micrometer-scale resolution, improving sensitivity and specificity. Zezza et al.^183^ developed a label-free optical biosensor for use in point-of-care (POC) testing. The fabrication involved the synthesis of acrylamide/propargyl acrylate (AM/PA) and AM hydrogels using free-radical polymerization with either photochemical or thermal activation. Surface micropatterns were created by using direct laser interference patterning on polyethylene terephthalate (PET) masters and then transferred to hydrogels via replica molding. The micropatterns allowed the hydrogel to diffract light, which was measured using a laser and photodiodes to detect changes during binding events. In the label-free hybridization assay, the micropatterned hydrogels were covalently functionalized with a specific thiol-modified oligonucleotide probe to detect different concentrations of a target DNA sequence. The obtained results showed a direct correlation between the diffraction efficiency measured and target concentration. Additionally, the micropattern allowed the system to detect the presence and concentration of the target without the need for additional labels or markers, improving the efficiency, accuracy, and consistency of the results. Regato-Herbella et al.^184^ synthesized thioether-based reactive oxygen species (ROS)-responsive difunctional monomers from EG/thioether acrylate (EG_n_SA) by a thiol-Michael addition click reaction. The resulting hydrogels exhibited photopolymerizable properties and were compatible with digital light processing 3D printing, enabling the fabrication of needles used as matrices for encapsulating 5-fluorouracil (5FU). This approach allowed the controlled release to be modulated not only by the presence of H_2_O_2_ but also by the enlarged surface morphology. Before proceeding to the next section, we briefly summarize some of the previously discussed examples of polymer-based hydrogels with biosensing applications (Table 1).

**Table 1:** Representative Examples of Polymer-Based Hydrogels for Biosensing

polymer type	materials	functionalization method/agent	bioreceptor	application in biosensing	ref
natural	agarose	Ag NWs-PBTMB	GOx	wearable flexible hydrogel patch for the noninvasive detection of physiological markers. Glucose was detected (0.02–5 mmol/L)	^66^
	chitosan	Apt-AgNPs	*E. coli* aptamer	SERS aptasensor for detection of pathogens (detection of *E. coli* with a linearized detection range from 3.2 × 10^1^ to 3.2 × 10^7^ CFU/mL)	^70^
synthetic	PEG	copolymerization with DHEBA	IgG antibody	hydrogel-based microparticles capable of detecting human IgG (0.1–1000 pM)	^77^
	PAA	CLC_solid_	MC aptamer	optical photonic biosensor for the detection of microcystin (MC)	^86^
	PNIPAM and PAM	Fe_3_O_4_@SiO_2_ CPCs	penicillinase	RPH-based optical biosensor for sensitive and colorimetric detection of a typical β-lactam PG (2.8 μM–2.8 mM)	^84^
hybrid and semisynthetic	CMCHI and OCMC	AuNCs	GOx	self-healing hydrogel for glucose detection (100 μM–5 mM) for in vivo and in vitro applications	^168^
	sodium alginate	5% CaCl_2_	anti-HER2 and anti-CA125 IgG antibodies	porous calcium alginate hydrogel immune beads for cancer detection (LOD of 0.004–0.005 ng/mL)	^170^
	CHI and HEC	AuNCs	cortisol specific aptamer	electrochemical salivary cortisol biosensor (0.1–50 ng/mL)	^45^
conductive	PANI	copolymerization with PA	carboxyl-functionalized DNA probes	electrochemical biosensor for MiRNA24 detection (1.0 fM–1.0 pM)	^126^
	PEDOT/PSS	PBNPs	GOx	electrochemical biosensor for noninvasive, continuous on-body monitoring of glucose in ISF (1–243 μM)	^132^
	PPy	CNTs	GOx	salivary glucose biosensor (linear- range detection of 100–700 μM)	^136^

## 4 Emerging Trends in Chemical Sensing and Biosensing

Despite the significant advances achieved with hydrogel-based biosensors, several challenges continue to limit their practical implementation, including insufficient sensitivity for ultralow analyte concentrations, limited multiplexing capabilities, signal attenuation in complex biological media, and difficulties in achieving continuous, real-time monitoring. In response, emerging sensing platforms are increasingly combining hydrogels with advanced electronics and increasingly combining hydrogels with advanced electronic and microfluidic technologies to enhance signal transduction, analytical performance, and device integration. Approaches such as hydrogel-gated field-effect transistors (FETs), organic electrochemical transistors (OECTs), conductive bioelectrodes, wearable microneedle systems, and lab-on-hydrogel devices leverage the unique physicochemical properties of hydrogels while overcoming many of the limitations of conventional sensing platforms. These developments are opening new opportunities for highly sensitive, multiplexed, miniaturized, and wearable biosensors for health care, environmental monitoring, and POC diagnostics.

### 4.1 Hydrogel-Gated FETs

Field-effect-transistor (FET) technology is gaining special interest in the biosensing research because of its potential for miniaturization, parallel sensing, and fast-response time.^185,186^ FET-based biosensors involve the integration of biorecognition molecules, where their interaction with bioanalytes could result in a biophysical or biochemical change that can further be transduced and amplified via the field effect toward external signal readouts.^187^ Additionally, nanomaterial-based FETs offer remarkable ability to detect biorecognition events without the need of redox mediators or electroactive labels. However, the existing close proximity between the bioreceptor and the FET channel/electrolyte interface via physical absorption or chemical conjugation could be critical for the “output” signal detection, a limitation known as the Debye length.^188^ This problem can be circumvented by integrating the bioreceptor in a hydrogel network, where the 3D matrix increases the effective Debye length and enhances the signal.^188,189^ For instance, Parichenko et al.^189^ implemented a PEG-heparin hydrogel hosting antibodies targeting the SARS-CoV-2 spike protein on silicon nanomaterial-based FETs and demonstrated their capability to monitor the disease progression in high ionic strength solutions through quantitative measurements (Figure 7a). Biosensing response was assessed in increasing concentrations of spiked samples, as well as in real viral samples. In both cases, the hydrogel could detect low concentrations (ranging from 5 to 50 pg/mL) of SARS-CoV-2 antigen and overcame the Debye length limitation. Although further improvement of the device may be needed due to the requirement of incubation–washing cycles and saturation issues, this hydrogel holds a significant potential in the anticipation of several diseases.^189^ In another example, Luo et al.^18^ developed a novel breath alcohol sensor based on a hydrogel-gated field-effect graphene transistor (HGGT). The oxidation of ethanol molecules by alcohol oxidase (AOx), generates H_2_O_2_ that is further electro-oxidated on the gate electrode of HGGT facilitated by carbon nanosheets (CNs). The resulting gate voltage shift is amplified by the field effect to a significant channel current change. The AOx/CNs-functionalized HGGT demonstrated superior selectivity by showing a large current response to 300 μM alcohol (34.61 ± 3.80 mV), but smaller responses to 1 mM glucose (1.83 ± 0.34 mV), among other tested analytes, as well as overcame the limitations of traditional methods, which include high cost for manufacturing, low accuracy, poor selectivity, and slow response.^190,191^

**Figure 7:**
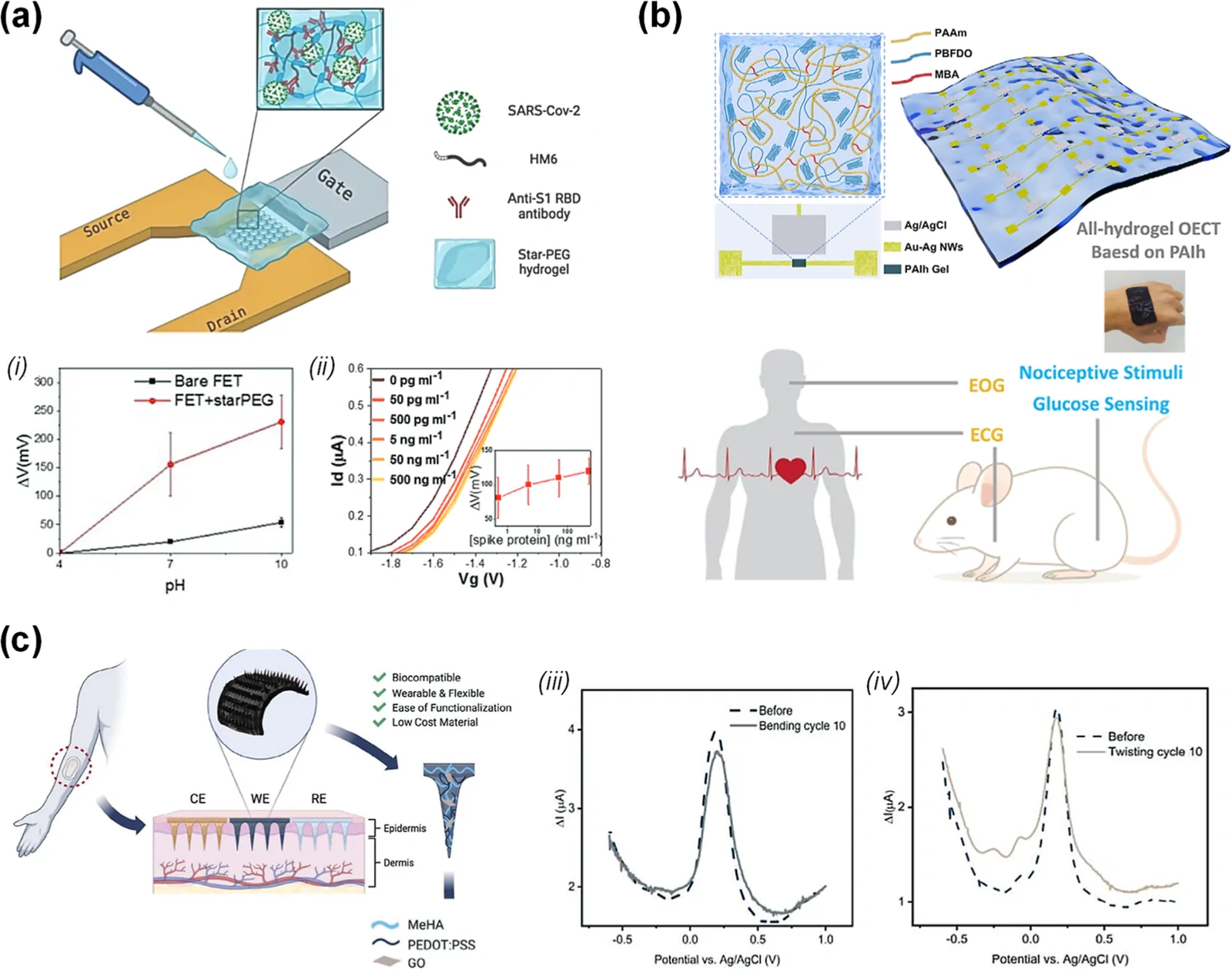
Emerging trends in chemical sensing and biosensing. (a) Schematic illustration of SARS-Cov-2 FET biosensing concept. HM6 corresponds to heparin maleimide 6. (i) pH sensitivity with and without gel. (ii) Response to the spike protein receptor binding domain in saliva, with the calibration graph as the inset. Reproduced with permission from ref 189 Copyright 2023 Wiley. (b) Schematic illustration of the PAIh semiconductor hydrogel structure and the hydrogel-based OECTs (top). Application of PAIh-based hydrogel OECTs in implantable biosignal monitoring (bottom). Reproduced with permission from ref 194 Copyright 2025 Wiley. (c) Schematic representation of MeHA PP-GO HMN as the working electrode in a three-electrode electrochemical sensor setup. The counter electrode is composed of gold-coated epoxy MNs, and the reference electrode is composed of Ag/AgCl-coated epoxy MNs. Graphs (iii) and (iv) show square wave voltammetry scans before and after 10 cycles of bending and twisting, respectively, proving a minimal impact on the average electrochemical signals. Reproduced with permission from ref 198 Copyright 2023 Wiley.

### 4.2 Hydrogel-Gated Organic Electrochemical Transistors (OECTs)

Biosensors based on organic electrochemical transistors (OECTs) are considered a promising platform for the development of highly flexible, sensitive, and wearable biosensors.^185^ In particular, research in the use of PEDOT:PSS in organic electrochemical transistors (OECTs) is also gaining increasing attention.^192^ PEDOT:PSS is considered as the most promising conductive polymer used as an active channel layer due to its high electrical conductivity, biocompatible properties, and stability in an aqueous environment.^193^ For instance, Jo et al.^193^ developed conductive PEDOT:PSS gel fibers with applications to wearable and implantable OECTs for biomedical fields. Conductive PEDOT:PSS microfibers were prepared by fibrillary gelation with a biocompatible choline-based IL, resulting in the formation of dense fibers with constant diameter by physical cross-linking from ionic interaction between PEDOT:PSS and ILs. A series of experimental studies of the physical and electrochemical properties of the formed fibers demonstrated their improved electric/ionic conductivity (400 S cm^–1^) and volumetric capacitance (154 F cm^–1^) of electronic devices. More specifically, this research highlights the potential of this approach for hydrogel-based OECTs, introducing a suitable candidate for applications in biointegrated electronics.^193^ Liang et al.^194^ focused on the development of an all-hydrogel-based OECT as a biocompatible and flexible platform for implantable physiological monitoring. Researchers fabricated a novel n-type depletion-mode semiconductor hydrogel made from poly­(benzodifurandione)/polyacrylamide/IL (PAIh), formed via IL-mediated phase separation, which enables efficient ion-electron coupling and tissue-like softness (Figure 7b). The developed device exhibited high transconductance (43 mS), mechanical flexibility (stable over 1500 bending cycles), high stability (physiological signals remained stable after 1 week of implantation), and excellent biocompatibility (>99% viability for fibroblasts and macrophages), with no cytotoxicity, inflammatory response, or oxidative stress observed in vitro and in vivo. In addition, this device was able to not only successfully amplify and record weak bioelectrical signals subcutaneously in rats but also detect glucose, through the development of an electrochemical glucose biosensor by immobilizing GOx in the OECT channel via glutaraldehyde cross-linking.

### 4.3 Hydrogel-Gated Bioelectrodes

Wearable flexible biosensors used for healthcare monitoring are gaining special attention not only within the scientific community but also among the general public.^195^ These smart wearable sensors can monitor the wearer’s health and surrounding environment in real time. Moreover, they can be physically attached to different parts of the human body and measure different parameters such as skin conductance, heart rate, changes in pH and glucose, etc.^195^ Regarding the great variety of health biomarkers in ISF and its highly similar composition compared to that of blood plasma,^196^ biosensors have gained significant interest as a noninvasive approach for monitoring these biomarkers.^197^ Interestingly, hydrogel-forming microneedles (HFMs) are minimally invasive devices that can be inserted into the tissue, access the ISF, and facilitate the diffusion of biotargets due to the swelling of their structure.^197^ In fact, it is important to highlight that solid microneedles can lead to issues such as skin irritation, whereas HFMs are formed of biocompatible materials.^198^ For biosensing applications, these hydrogels can be functionalized previously with sensing elements capable of detecting an specific target on biofluids, and their high resolution fabrication in a fast and low-cost manner through 3D-printing is gaining increasing interest.^199^ For instance, Shirzadi et al.^198^ developed a versatile electrode using a biocompatible PEDOT:PSS-based composite hydrogel for wearable microneedle sensing platforms to analyze ISF. The researchers fabricated the hydrogel-based microneedle electrodes by combining PEDOT:PSS, as the conductive polymer, methacrylated hyaluronic acid (MeHA), as the hydrogel backbone, and GO, not only as the filler to improve the mechanical strength of the electrodes but also to enable the immobilization of biomolecules (Figure 7c). The mechanical compression tests performed on the hydrogels confirmed their sufficient robustness for successful insertion into the skin. It also showed sufficient swelling capacity for extracting ISF and facilitate sensing. Furthermore, results from an ex vivo porcine skin model assay showed that the skin’s deformation and movement had a minimal impact on the electrochemical signals, proving the potential of HMN electrodes for in vivo measurements. Kim et al.^200^ developed a robust electrochemical biosensor coupled with a microneedle-based ISF extraction system for noninvasive cholesterol monitoring. The microneedle was based on an acrylamide-based hydrogel network, and the cholesterol biosensing system was based on a combination of a graphene-based electrode and a PANI interlayer with immobilized ChOx. Researchers injected the microneedle into a cholesterol-absorbed porcine skin to extract ISF. After the removal of the microneedle from porcine skin, ISF was extracted and measured by the fabricated electrochemical biosensor. The obtained results demonstrated that this fabricated sensing technology was capable of specifically monitoring cholesterol (0.2–100 mg/dL) from extracted ISF with high sensitivity and reliability. Although conductive HFMs have been recently reported toward a variety of biosensing and drug delivery applications,^201–204^ future research, such as tests on the skin of living human subjects, is essential for the development of smart wearable HFM devices for real-time monitoring.^200^

Conductive hydrogels play a key role in the development of these wearable devices, especially CPHs.^32^ Despite the evidential progress in the field of conductive hydrogel-based wearable biosensors, many hydrogels used water as the dispersing medium, which sometimes resulted in water loss and phase separation after a long period of time, swelling in extreme humidity conditions, or even freezing at subzero temperatures.^205,206^ These characteristics would significantly disrupt their electrical conductivity and mechanical performance, limiting their practical application in different environments.^207^ Among the different candidates to eliminate the problems of freezing and dehydration in hydrogels, DES have attracted special interest because of their excellent thermal stability, nonvolatility, nontoxicity, low cost, and good biocompatibility.^206^ DES are defined as a mixture of two or more compounds for which the eutectic point temperature is lower to that of an ideal liquid mixture.^208^ Although DES are a subclass of ILs, compared to this group, they are cheaper and easier to produce, less toxic, and often biodegradable.^209^ Recently, the research on the construction of DES into a gel to generate a “eutectogel” has significantly increased. For instance, Sun et al.^206^ developed a PVA/SA/MXene network hydrogel dissolved in DES. MXene functioned as the two-dimensional conductive filler, and DES as the antioxidant dispersing medium. The DES mixture was composed of choline chloride (ChCl) and AA in a 1:2 molar ratio. The prepared eutectogel demonstrated excellent mechanical properties, high ionic conductivity (42.84 μS/m), good biocompatibility (>97% cell viability), rapid detection time (400 ms), high strain sensitivity (GF = 7.66), and wide temperature tolerance (−20 to 60 °C). The use of DES instead of water not only improved the antifreeze and solvent retention properties but also conferred antimicrobial and self-adhesive properties.^206^

### 4.4 Lab-On-Hydrogel Platforms for Multiplexed Sensing

Regarding the success of lateral flow assays in the rapid detection of biomarkers, the development of diagnostic technologies for clinical applications is essential. Hydrogels are gaining increasing attention in the development of diagnostic platforms, due to their higher biocompatibility, solution-like kinetics, and nonfouling nature, compared to surface-based biomolecule detection systems.^210,211^ As an example, Juthani and Doyle^210^ presented a hydrogel-based multiplexed colorimetric platform which could be imaged using a cell phone. Briefly, a reporter enzyme (streptavidin-enzyme conjugate), which catalyzed the production of a chromogenic precipitate in the presence of its substrate, was encapsulated in the 3D network of a hydrogel microparticle. This method of precipitation-based colorimetric detection was employed for microRNA (miRNA) detection, an emerging class of biomarkers that are dysregulated in many diseases, such as cancer or Alzheimer’s.^212^ Briefly, the entrapped enzyme attached to the miRNA produced a blue indigo precipitate that stayed trapped within the hydrogel’s 3D network due to its porous structure and hydrophobic environment, preventing diffusion. This localized color change enabled the multiplexed detection of miRNAs (LOD of 0.22 fmol), and its possible application to other biomolecules such as protein detection. In addition, Morioka et al.^213^ proposed an analytical platform formed of a compact fluorescence imager and a microfluidic chip equipped with functional hydrogels. In particular, the multilayered structure of the agarose-based hydrogel functioned, on the one hand, as a separation gel of contaminant proteins from glucose (the model analyte), and on the other hand, as a reaction gel in which the fluorescence intensity was proportional to the glucose concentration through an enzymatic reaction. Its analytical performance was demonstrated by measurement of glucose in human serum, with an LOD of 21 μM and a rapid detection time (5 min). This research had the aim to overcome the problems of instrument miniaturization and interference, through the development of a Lab-on-gel device equipped with agarose gels of different compositions to integrate preprocessing and sensing.

## 5 Conclusions and Perspectives

Among the diverse hydrogel systems investigated for biosensing, polymer-based hydrogels have emerged as the most versatile and widely adopted platforms due to their unique combination of physicochemical tunability, biocompatibility, conductivity, and multifunctionality.^42^ Unlike inorganic or low-molecular-weight gel systems, polymer hydrogels offer highly customizable network architectures that allow for precise control over mechanical strength, porosity, swelling behavior, degradation kinetics, and bioactive interactions. By combining target-specific bioreceptors with advanced functionalization methods, polymer-based hydrogels offer a versatile and tunable platform for the development of smart and next-generation biosensors with applications in medical diagnostics, environmental monitoring, and wearable and implantable technologies. Natural polymers such as alginate, gelatin, collagen, and chitosan provide intrinsic biocompatibility and extracellular matrix-mimicking characteristics, facilitating cell interaction, biomolecule stabilization, and efficient analyte transport, whereas synthetic polymers including PAM, PEGDA, HEMA, and PVA contribute superior structural stability, reproducibility, and processability. Moreover, intrinsically conductive polymers such as PANI, PPy, and PEDOT and their derivatives provide efficient charge transport pathways while preserving the soft, hydrated, and biocompatible nature of the hydrogel matrix. Recent studies have demonstrated that advances in precision polymer-based hydrogel engineering like the incorporation of conductive nanofillers, bioactive molecules, and stimuli-responsive moieties have enabled the design of hydrogels with application-specific functionalities. Collectively, these characteristics position polymer-based hydrogels as a uniquely advantageous class of materials that bridge biosensing, wearable electronics, and personalized healthcare, explaining their dominant role in the development of next-generation biosensor technologies.

Despite the promising potential of polymer-based hydrogels as the material of choice for the development of innovative biosensing devices, few of them exhibit all the requirements to act as wearable, flexible, injectable, and conductive polymeric hydrogel-based biosensors.^104^ Figure 8 illustrates the critical relationship between the established material properties of polymer-based hydrogels and the future trends that must be explored to overcome existing limitations. Therefore, ongoing research continues to address some challenges in developing multifunctional polymer-based hydrogels for biosensing applications, as well as their clinical adoption and commercial deployment.^119,122^ One of the main challenges may be the reproducibility and scalability related to the production of these hydrogels at industrial levels, as well as the simplification of manufacturing processes without compromising functionality. Hydrogels are highly tunable materials, but many reported sensor platforms rely on complex synthesis routes, multistep functionalization procedures, or customized fabrication techniques that are difficult to reproduce at an industrial scale. Batch-to-batch variations in polymer composition, cross-linking density, swelling behavior, and mechanical properties can significantly affect sensing performance, resulting in limited manufacturing consistency and quality control. Achieving standardized and reproducible fabrication protocols is, therefore, essential for commercialization.

**Figure 8:**
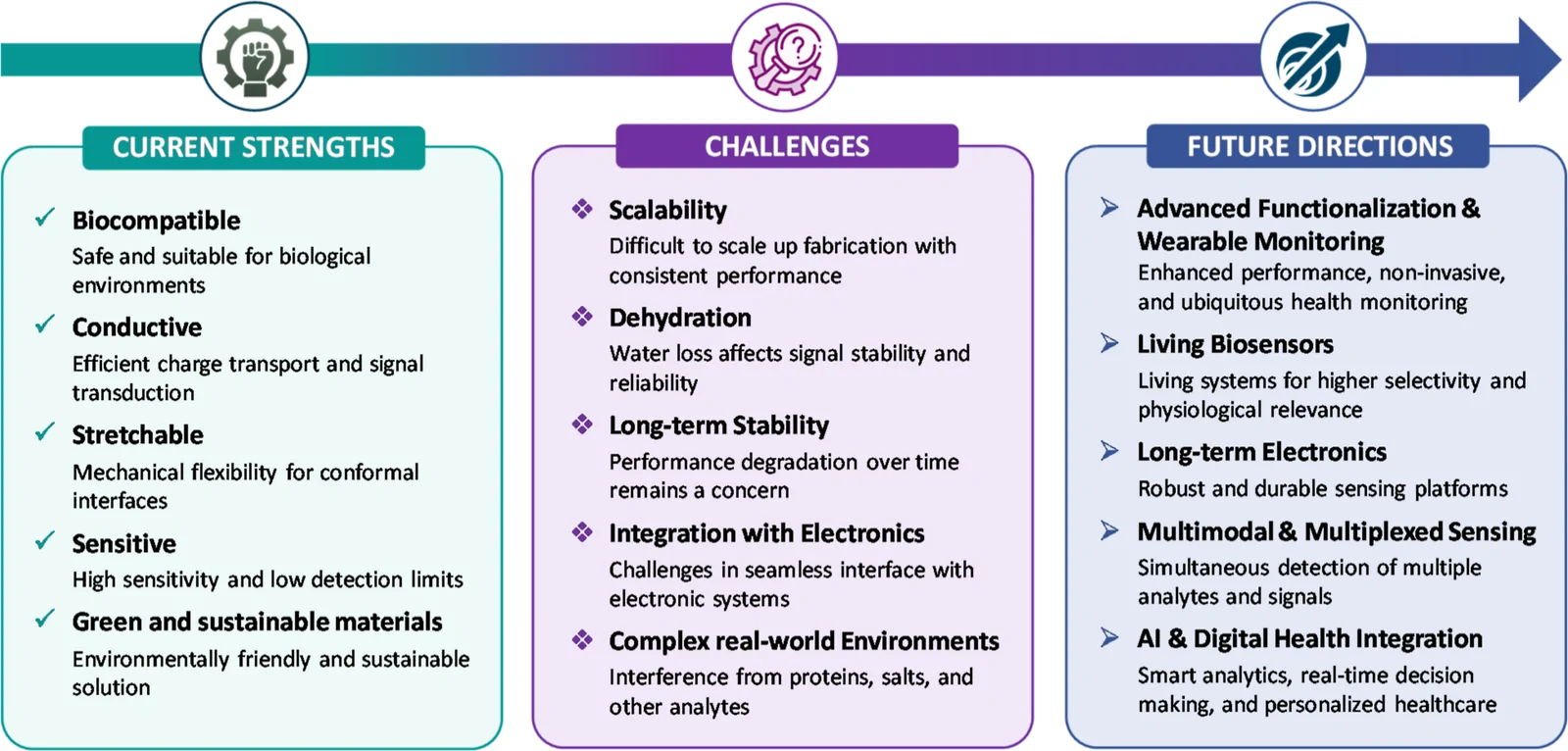
Comprehensive overview of polymer-based hydrogels for chemical biosensing, highlighting current advances, existing challenges, and future research directions.

Furthermore, it is essential to achieve the required long-term stability and biocompatibility for prolonged use in wearable applications.^214^ Although hydrogels generally exhibit favorable tissue compatibility due to their high-water content and extracellular matrix-like characteristics, concerns remain regarding cytotoxicity of cross-linking agents, degradation byproducts, residual monomers, and incorporated conductive nanomaterials.^215^ Dehydration may be also an issue in real-word applications, as hydrogels lose water content in dry environments, resulting in lower ionic conductivity and mechanical stiffness. Maintaining reliable sensor function over extended periods requires optimization of hydrogel chemistry, mechanical robustness, antifouling properties, and environmental stability. From a regulatory perspective, successful clinical translation requires rigorous validation of safety, reproducibility, manufacturing quality, and analytical accuracy under real-word conditions.^216^ Future research should therefore move beyond proof-of-concept demonstrations and place greater emphasis on standardized manufacturing processes, Good Manufacturing Practice (GMP)-compatible production methods, long-term biocompatibility assessment, and large-scale clinical validation. Addressing these challenges will be crucial for transforming hydrogel-based biosensors from promising research prototypes into clinically approved and commercially viable diagnostic platforms. It is noteworthy that owing to the versatility of hydrogel-based electrochemical biosensors, they can also be employed for other applications in different research fields that are closely related to human health. For example, Gao et al.^217^ fabricated a miniaturized chitosan hydrogel-based electrochemical biosensor for determination of heavy metal ions and phenolic compounds in wastewater, by using yeast cells as the signal receptor. *Saccharomyces cerevisiae* cells were coimmobilized with potassium ferricyanide-menadione as mediators on a chitosan hydrogel polymer film with boron-doped nanocrystalline diamond (BND) particles electrodeposited onto a glassy carbon (GC) electrode. BND particles improved the conductivity of the device, and the detection of toxicity of wastewater samples was successfully performed.^217^ Cell-based hydrogel biosensors are also a promising tool for the detection of biotoxicity in complex water samples. Gao et al.^217^ fabricated a double-mediator based whole cell electrochemical biosensor for the assessment of acute biotoxicity in wastewater. For the development of the device, *S. cerevisiae* cells were encapsulated in a chitosan-based hydrogel polymer film with BND particles which was electrodeposited onto a GC electrode. The rapid and highly sensitive electrochemical response of biological cells was enhanced by employing a double-mediator strategy, involving menadione and potassium ferricyanide. Menadione facilitated electron transfer from the encapsulated yeast cells to the electrode. On the other hand, potassium ferricyanide acted as a redox mediator that enhances the electrochemical signal by interacting with the electrons released from the metabolic activity of the cells. The inhibition rates of yeast metabolic activity observed for the tested wastewater samples demonstrated the applicability of the biosensor for real-time biotoxicity assessment in environmental monitoring. Despite evidential efforts into the development of cell-based biosensors,^218–220^ cell-based conductive hydrogel-based biosensors represent an emerging area to explore, and further research is necessary to exploit their potential in the biosensing field.
